# Determination of the peroxisomal proteome of pathogenic stage *Histoplasma capsulatum*

**DOI:** 10.1128/mbio.00771-26

**Published:** 2026-06-09

**Authors:** Peter J. Brechting, Chad A. Rappleye

**Affiliations:** 1 Department of Microbiology, Ohio State University 2647, Columbus, Ohio, USA; University of Melbourne, Melbourne, Victoria, Australia

**Keywords:** *Histoplasma*, peroxisome, proximity labeling, proteomics, pathogenesis, virulence determinants, siderophores

## Abstract

During infection, *Histoplasma capsulatum* exists primarily as an intracellular pathogen of phagocytes, where it overcomes host defense mechanisms and replicates within the phagosome. This intracellular proliferation of *Histoplasma* yeasts requires the function of the fungal peroxisome for multiple, incompletely defined roles, with early pathogenesis functions dependent on the proper trafficking of peroxisomal matrix proteins carrying a type 1 peroxisome-targeting signal (PTS1). To better understand the potential roles of peroxisomes during *Histoplasma* infection, we identified the constituents of the peroxisomal proteome of pathogenic-phase *Histoplasma* yeasts through proximity labeling using a peroxisome-targeted TurboID biotin ligase. Comparative proteomics using peroxisome- or cytosol-localized TurboID identified 90 high-confidence peroxisomal proteins. To validate the peroxisomal proteome, a subset of nine putative peroxisomal proteins was localized to the peroxisome using fluorescent protein fusions. Comparison of peroxisomal proteomes for *Histoplasma* yeasts lacking the Pex5 or Pex7 cytosolic PTS1 or PTS2 receptors assigned proteins to either the PTS1- or PTS2-dependent peroxisomal import pathways, and refined the Histoplasma PTS1 tripeptide consensus sequence. The peroxisomal proteome of *Histoplasma* yeast during macrophage infection was ultimately determined to provide relevance to *Histoplasma*’s primary pathogenic niche. The identities of the proteins comprising the peroxisomal proteome include several enzymes for canonical peroxisome-localized biochemical pathways (e.g., fatty acid utilization and siderophore biosynthesis), but also represent several potential novel functions for the organelle. The defined peroxisomal proteome of pathogenic-phase *Histoplasma* yeasts, particularly the set of proteins imported via the PTS1 import pathway, thus establishes the important foundation for understanding how peroxisomes promote intracellular pathogenesis.

**IMPORTANCE:**

Peroxisomes are eukaryotic organelles that serve as a subcellular compartment for diverse metabolic and biosynthetic reactions, some of which play crucial roles in supporting the intracellular pathogenesis of the fungal pathogen *Histoplasma capsulatum*. In this study, we used proximity labeling with the biotin ligase TurboID to capture peroxisomal matrix proteins, enabling the empirical derivation of the peroxisomal proteome of pathogenic-phase *Histoplasma* yeasts, including during *Histoplasma* infection of mammalian cells. This represents the first organelle-specific proteome definition in *Histoplasma* and highlights the utility of proximity-based biotinylation and comparative proteomics for understanding how subcellular compartments and their functions contribute to intracellular pathogenesis.

## INTRODUCTION

*Histoplasma capsulatum* is a dimorphic fungal pathogen of mammals and the causative agent of histoplasmosis. *Histoplasma* is endemic to the Mississippi and Ohio River valleys as well as broad sections of Latin and South America, and there is a growing number of histoplasmosis cases seen worldwide (1, 2). In the environment, *Histoplasma* grows in a filamentous (mycelial) form, which produces airborne conidia. Inhalation of the conidia by a mammal and exposure to mammalian body temperature causes the *Histoplasma* conidia to germinate into the virulent yeast phase (3, 4). Within the mammalian host, *Histoplasma* yeasts reside predominantly in the phagosomal compartment of alveolar macrophages and other phagocytic cells (5, 6). During the innate immune stage of infection, the macrophage phagosome environment is permissive for *Histoplasma* proliferation, as *Histoplasma* can detoxify host-derived ROS species and prevent acidification of the phagosome (7–9). *Histoplasma* yeasts proliferate intracellularly by metabolizing available gluconeogenic carbon sources, synthesizing essential vitamins, and acquiring micronutrients such as metals (10–15). However, the development of cell-mediated immunity and the consequent activation of phagocytes by Th1-type cytokines result in restriction of *Histoplasma* growth in the phagosome (16, 17). The host-microbe interactions that underlie these phagocyte dynamics and intracellular fungal growth are not fully understood. We previously identified a major role for the peroxisomes of *Histoplasma* during both stages of infection (18).

Peroxisomes are eukaryotic organelles whose biogenesis and maintenance are mediated by a suite of peroxin (Pex) proteins (19, 20). The diverse functions of the organelle are carried out by peroxisomal matrix proteins (21), which are synthesized in the cytosol and subsequently imported into the peroxisome lumen (21). The majority of soluble matrix proteins are targeted to the organelle via a type 1 peroxisome-targeting signal (PTS1) located at the extreme C-terminus of the protein (22, 23). The peroxin protein Pex5 acts as a cytosolic receptor that binds the PTS1 motif and facilitates the transfer of PTS1-containing proteins to the peroxisomal import machinery in the organelle membrane. In an analogous manner, soluble proteins which contain a type 2 peroxisome-targeting signal (PTS2) near the N-terminus are bound by the cytosolic Pex7 protein, which delivers the PTS2 matrix proteins to the peroxisomal import machinery (20, 23, 24). *Histoplasma* pathogenesis requires both Pex5- and Pex7-dependent matrix protein import, indicating that both PTS1- and PTS2-containing peroxisomal matrix proteins contribute to *Histoplasma* virulence (18). However, the specific peroxisome functions that facilitate *Histoplasma’s* successful infection of phagocytes remain largely unknown.

Peroxisomes are functionally diverse between species but often serve as a subcellular compartment for specific metabolic pathways. This compartmentalization of these functions is thought to benefit the cell by sequestering toxic byproducts and intermediates, increasing local concentrations of substrates, preventing competition with other enzymes, or regulating enzymatic activity by alteration of redox balance (21, 25). In most cells, peroxisomes contain enzymes for β-oxidation of long- and medium-chain fatty acids and enzymes of the glyoxylate shunt that enable metabolic utilization of fatty acids and two-carbon compounds as carbon sources. Localization of a peroxisomal catalase, peroxiredoxins, and glutathione reductase to the peroxisome detoxifies ROS generated by many of the peroxisomal reactions. Peroxisomes also play a key role in the biosynthesis of several secondary metabolites, including the vitamin biotin (26, 27), several mycotoxins—such as aflatoxin, gliotoxin, and AK toxin (28–30)—and iron-scavenging siderophores (18, 31, 32).

The peroxisomes of *Histoplasma* yeasts play multiple roles in *Histoplasma* pathogenesis as disruption of peroxin proteins attenuates *Histoplasma* virulence (18). The PTS1 and PTS2 peroxisomal protein import pathways provide distinct virulence functions: loss of PTS1-protein import in Pex5-deficient yeasts attenuates *Histoplasma* virulence during the early stages of infection, while loss of PTS2-protein import in Pex7-deficient yeasts only impairs *Histoplasma* virulence after activation of cell-mediated immunity (18). Interestingly, depletion of canonical functions of the peroxisomes does not recapitulate the attenuated virulence of peroxisome function-deficient yeasts; the β-oxidation and glyoxylate shunt enzymes are dispensable for *Histoplasma* during infection (15), and a biotin auxotroph strain of *Histoplasma* retains full virulence (12). Furthermore, loss of the peroxisomal catalase does not increase fungal susceptibility to host-derived ROS, due to the expression of a redundant catalase (8). Our previous work indicated that *Histoplasma* biosynthesis of hydroxamate siderophores is partially localized in peroxisomes and dependent on both the PTS1- and PTS2-import pathways (18). Siderophore biosynthesis is necessary for *Histoplasma* growth in iron-limiting conditions, but the loss of siderophores or import of PTS2-peroxisomal matrix proteins reduces virulence only after activation of cell-mediated immunity. In contrast, loss of PTS1-peroxisomal matrix protein import attenuates *Histoplasma* virulence during the early stages of infection (18). Thus, the peroxisome plays a non-siderophore-related role that facilitates intracellular growth of *Histoplasma*, and PTS1-containing matrix proteins contribute an additional function(s) necessary for *Histoplasma* virulence.

Identification of candidate peroxisome functions that promote *Histoplasma* virulence requires defining the repertoire of peroxisomal matrix proteins in pathogenic *Histoplasma* yeast cells. Proteomic analysis of peroxisomes isolated via subcellular fractionation has identified matrix proteins of *Saccharomyces* and *Penicillium* peroxisomes (33–35), and high-throughput microscopy screens for peroxisomal targeted proteins have expanded the understanding of the organelle’s protein content in *Saccharomyces cerevisiae* (36). However, both methods are difficult or impossible to implement in less tractable systems, particularly non-model organisms such as *Histoplasma*. Bioinformatic identification of proteins with canonical PTS sequences provides an alternate approach, but this *in silico* identification strategy can miss proteins targeted via non-canonical methods or proteins with poorly predicted gene models, and may mischaracterize proteins that are dually localized to peroxisomes and other compartments (37–39). Furthermore, reliance on bioinformatic catalogs ignores phase-specific (e.g., yeast phase) and infection-specific matrix protein production by dimorphic organisms such as *Histoplasma*.

In recent years, enzyme-catalyzed proximity labeling has provided an avenue for examining the spatial organization of proteins in live cells. This technique employs a promiscuous labeling enzyme that catalyzes the spatially restricted covalent binding of a small molecule, such as biotin, to nearby proteins, enabling their capture via streptavidin and subsequent identification of labeled targets (40, 41). To identify peroxisomal matrix proteins of pathogenic-stage *Histoplasma*, we used proximity labeling with the biotin ligase TurboID (40) to tag and capture peroxisomal matrix proteins, which were subsequently identified by mass spectrometry-based proteomics. To specifically label the peroxisomal matrix proteins, we took advantage of the spatial restriction of these proteins from the cytosol by targeting the TurboID biotin ligase to the organelle lumen. Furthermore, labeling of matrix proteins in PTS1- and PTS2-import pathway-deficient yeasts (Pex5- and Pex7-deficient yeasts, respectively) allowed empirical separation of the peroxisomal proteome members into specific import pathways. The functions represented by the PTS1-containing matrix proteins highlight candidate peroxisome roles that contribute to *Histoplasma* virulence during the early stages of infection (i.e., before cell-mediated immunity).

## RESULTS

### Targeting of TurboID to peroxisomes in *Histoplasma*

**Fig 1:**
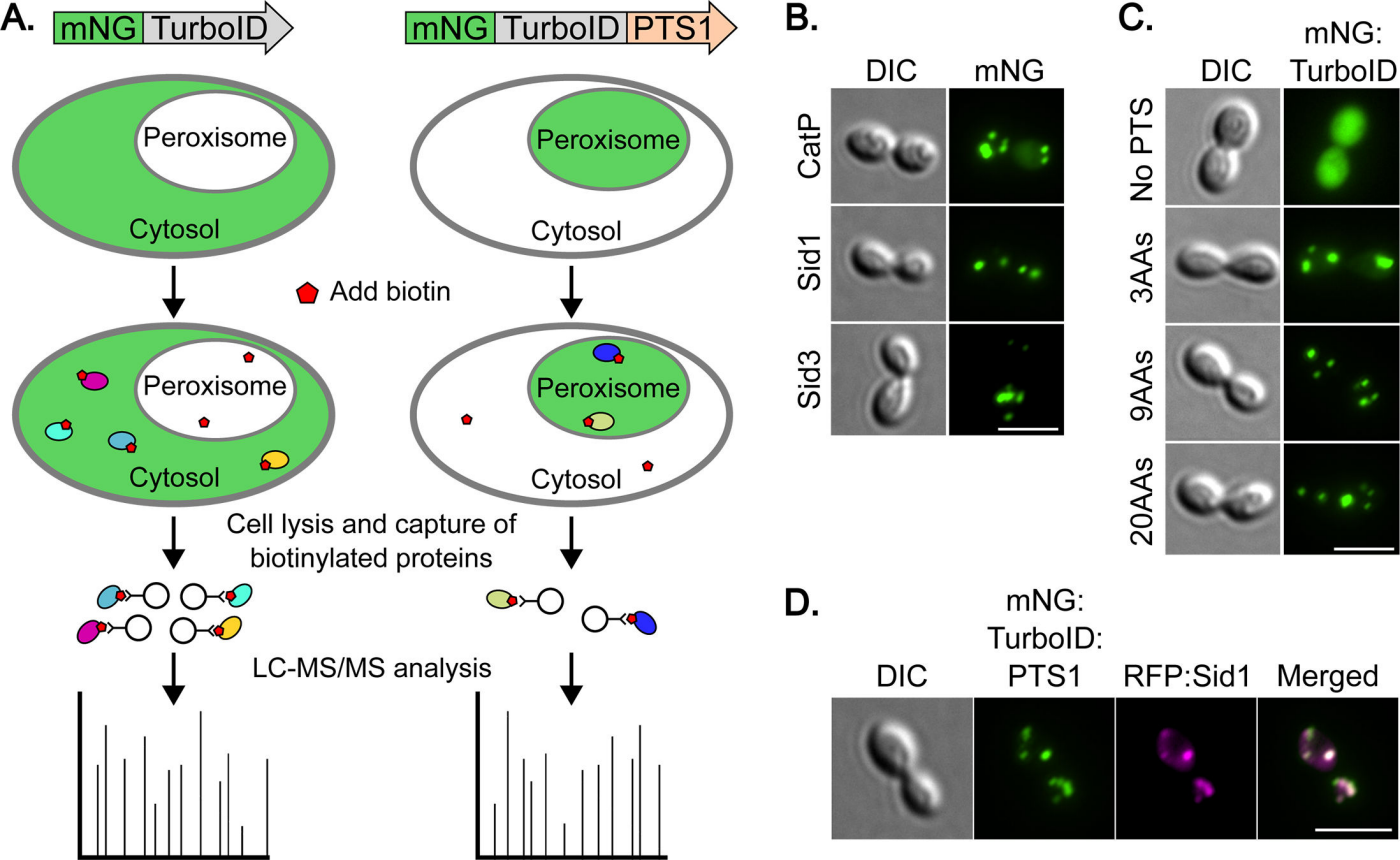
Localization of TurboID to *Histoplasma* peroxisomes for peroxisomal proteomics. (**A**) Strategy for proximity-dependent biotinylation experiments to capture peroxisomal matrix proteins. TurboID is fused to a monomeric NeonGreen (mNG) fluorescent protein and targeted to the peroxisome via the addition of a type 1 peroxisome-targeting signal (PTS1) at the C-terminus. Addition of exogenous biotin facilitates TurboID-dependent biotinylation of proteins within the same cellular compartment as TurboID. After labeling, cells are lysed and biotinylated proteins are captured via biotin-streptavidin interaction and identified via LC-MS/MS. (**B**) Localization of mNG harboring the PTS1 signals from the peroxisomal proteins CatP, Sid1, and Sid3. Images depict differential interference contrast (DIC) and fluorescence images of *Histoplasma* yeasts expressing mNG fused to the C-terminal 3 amino acids from the peroxisomal proteins CatP, Sid1, or Sid3. (**C**) Improved peroxisomal localization of TurboID with extended C-terminal PTS1 sequences. Microscopy images show localization of mNG:TurboID fusion constructs with the addition of C-terminal PTS1 sequences of increasing lengths (3, 9, or 20 amino acids [AA]) derived from the Sid3 protein. (**D**) Confirmation of peroxisomal localization of TurboID by the Sid3 C-terminal 20 amino acids. Microscopy images show localization of mNG:TurboID with the C-terminal 20 amino acids from Sid3 which co-localize with the peroxisome-localized red fluorescent protein (RFP) fused to the Sid1 protein. Scale bar represents 5 µm.

To identify the constituent proteins of the *Histoplasma* pathogenic-phase peroxisome, we utilized a comparative proximity labeling proteomics approach with the biotin ligase TurboID localized either to peroxisomes or the cytosol of *Histoplasma* yeast (Fig. 1A). TurboID was selected for these experiments due to its high catalytic activity and ease of use in fungi, as alternatives such as the ascorbate peroxidase APEX2 require the addition of biotin phenol, which is impermeable to yeast cells (42, 43). To identify the optimal peroxisome-targeting sequence for selective targeting of TurboID to the peroxisome in *Histoplasma*, three amino acid PTS1 sequences derived from established *Histoplasma* peroxisomal proteins, which fit the consensus derived from *Saccharomyces cerevisiae* ([A/S/C/P]-[K/R/H]-L), were appended to the C-terminus of the monomeric NeonGreen (mNG) fluorescent protein (22). The tripeptides examined were derived from the *Histoplasma* peroxisomal catalase CatP (-PRL) and the siderophore synthesis enzymes Sid1 (-ARL) and Sid3 (-SKL) (18). PTS1 sequences from each of these proteins directed the fluorescence of mNG to subcellular puncta representing peroxisomes (Fig. 1B), although the CatP PTS1 sequence also showed some cytosol-localized mNG fluorescence (44). To target TurboID to peroxisomes, the Sid3 PTS1 was added to the C-terminus of TurboID, and mNG was fused to the N-terminus to permit localization confirmation. To determine if amino acids before the Sid3 PTS1 tripeptide could provide more complete peroxisomal localization as has been seen in other organisms (44), we compared localization of mNG:TurboID with the C-terminus of Sid3 of different lengths. The C-terminal nine or 20 amino acids (-TAKPSKSKL or -VVNFGNVTSNGTAKPSKSKL, respectively) caused the most complete targeting of TurboID to peroxisome-like puncta (Fig. 1C). The strain expressing TurboID fused to the Sid3 C-terminal 20 amino acid sequence was selected for subsequent experiments. This mNG:TurboID:PTS1 construct co-localized with the known peroxisomal matrix protein Sid1 tagged with td-Tomato red fluorescent protein (RFP) (Fig. 1D), confirming peroxisomal localization of the PTS1-tagged TurboID.

### Optimization of TurboID-based proximity labeling conditions in *Histoplasma*

**Fig 2:**
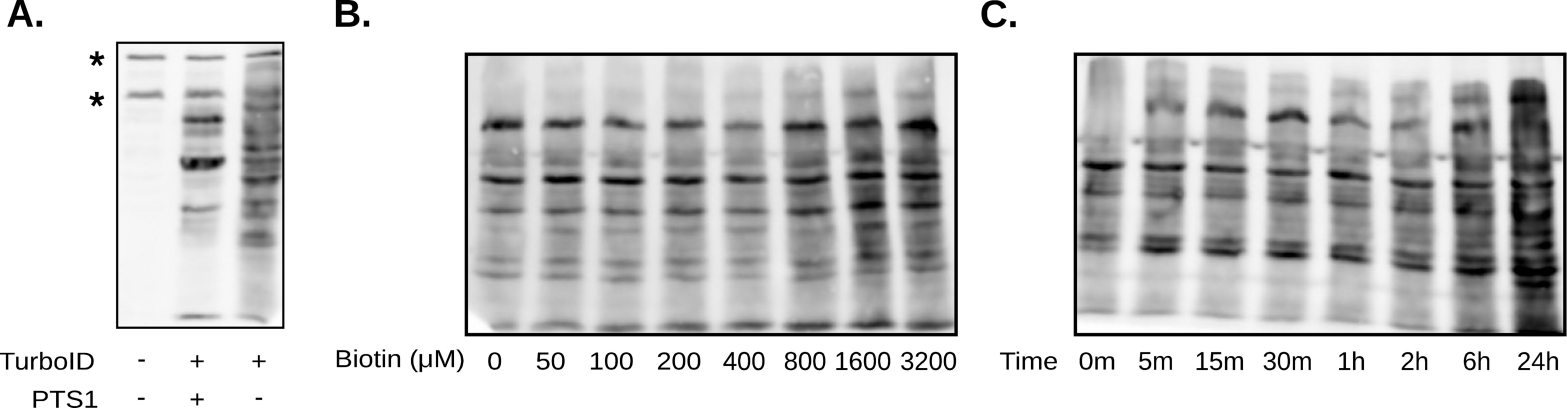
Expression of TurboID in *Histoplasma* results in biotinylation of proteins in a biotin- and time-dependent manner. (**A**) Distinct protein biotinylation patterns in cell lysates from cytosolic and peroxisomal-localized TurboID. *Histoplasma* yeasts expressing TurboID or TurboID with a PTS1 localization signal were exposed to 1 mM biotin, and biotinylation was visualized by electrophoretic separation of cellular lysates and detection by HRP-conjugated streptavidin. Asterisks indicate pyruvate carboxylase and 3-methylcrotonyl-CoA carboxylase, two endogenously biotinylated proteins. (**B and C**) Optimization of proximity labeling in peroxisomes of *Histoplasma* yeasts. TurboID:PTS1-expressing yeasts were incubated with increasing concentrations of biotin for 6 h (**B**) or with 1 mM biotin for varied durations (**C**), and protein biotinylation was assessed by streptavidin-based detection.

While TurboID has been used in fungi to define interacting proteins and compartment-specific proteins (42, 45, 46), TurboID has not been employed in *Histoplasma* nor used to identify any fungal peroxisomal matrix proteins. To confirm that TurboID could biotinylate proteins in pathogenic-phase *Histoplasma* yeasts, we examined the biotinylation of proteins in yeasts expressing the mNG:TurboID following the addition of exogenous biotin (Fig. 2A). In the absence of TurboID, two proteins were consistently biotinylated corresponding to the known endogenously biotinylated proteins pyruvate carboxylase and methyl-crotonyl-CoA carboxylase (47). After the addition of exogenous biotin, cell lysates from yeasts expressing mNG:TurboID had an increased number of biotinylated proteins, the electrophoretic profile of which depended on peroxisomal or cytosolic localization of TurboID (mNG:TurboID:PTS1 or mNG:TurboID, respectively) (Fig. 2A). These results confirm the biotinylation activity of mNG:TurboID and indicate the subcellular compartment-specific protein labeling by TurboID.

To maximize biotinylation for detection of low-abundance proteins, we determined the optimal labeling conditions for TurboID-based biotinylation in *Histoplasma* yeasts. First, we performed the TurboID-mediated labeling of cellular proteins with increasing amounts of exogenous biotin. Concentrations of added biotin above 800 µM showed abundant protein biotinylation, which did not significantly increase with greater biotin concentrations (Fig. 2B). Addition of 1 mM biotin was selected for optimal biotinylation. Next, we performed the TurboID-mediated labeling of cellular proteins with increasing incubation times with biotin. Incubation times ranging from 5 min to 2 h produced similar amounts of biotinylation (Fig. 2C). Prolonging the time to 6 h increased the number of biotinylated proteins, which further increased after 24 h of incubation. Despite the notable increase in protein biotinylation after 24 h, we selected 6 h as the optimal time for biotinylation of cellular proteins to avoid potential changes in cellular processes that could result from inactivation of proteins and enzymes by prolonged post-transcriptional modification by biotinylation.

### Comparative proteomics reveal peroxisomal proteins of *Histoplasma yeasts*

**Fig 3:**
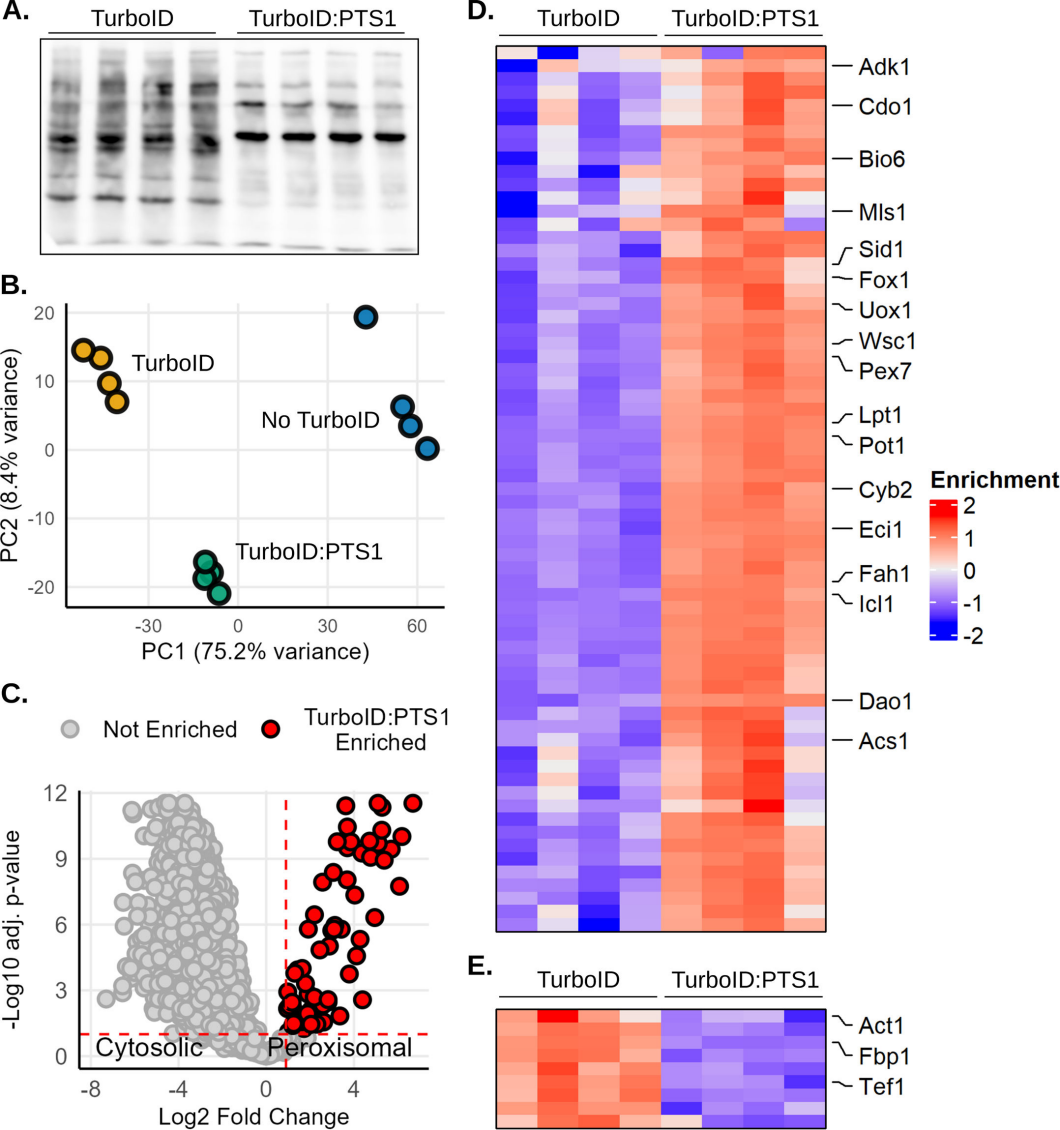
Proximity labeling with peroxisomal-localized TurboID reveals peroxisome-enriched proteins. (**A**) Total biotinylation of proteins from *Histoplasma* yeast expressing cytosolic mNG:TurboID (TurboID; lanes 1–4) or peroxisome mNG:TurboID:PTS1 (TurboID:PTS1; lanes 5–8). 5 × 10^9^ *Histoplasma* yeast cells were incubated with 1 mM biotin for 6 h. Compartment-specific biotinylation was assessed via probing proteins from cellular lysates with HRP-conjugated streptavidin after electrophoretic separation. (**B**) Principal component analysis (PCA) of biotinylated protein populations from the cytosol- and peroxisome-localized TurboID strains. *Histoplasma* yeasts lacking TurboID (“No TurboID”) or strains expressing TurboID or TurboID:PTS1 were incubated with 1 mM biotin, and the biotinylated proteins were captured with streptavidin-conjugated agarose resin. The captured proteins were identified by LC-MS, and the protein identities were clustered for PCA. (**C**) Separation of biotinylated proteins into peroxisome and cytosol-enriched protein populations. Proteins showing differential enrichment in lysates from TurboID:PTS1 yeasts (adjusted *P*-value < 0.1, fold change > 1.866) are indicated in red. (**D and E**) Clustering of significantly enriched peroxisomal proteins (**D**) or expected cytosolic proteins (**E**) after biotinylation with peroxisomal (TurboID:PTS1) or cytosolic TurboID. Identified biotinylated peroxisome-enriched proteins include adenylate kinase (Adk1), cysteine dioxygenase (Cdo1), KAPA synthase (Bio6), malate synthase (Mls1), L-ornithine monooxygenase (Sid1), fatty-acyl coenzyme A oxidase (Fox1), urate oxidase (Uox1), woronin sorting complex protein (Wsc1), peroxin 7 (Pex7), lipid transfer protein (Lpt1), 3-ketoacyl-CoA thiolase (Pot1), L-lactate cytochrome-c oxidoreductase (Cyb2), Δ3, Δ2-enoyl-CoA isomerase (Eci1), fumarylacetoacetate hydrolase (Fah1), isocitrate lyase (Icl1), D-amino acid oxidase (Dao1), and acetyl-coenzyme A synthetase (Acs1). Biotinylated proteins captured in the cytosol include the metabolic proteins (glyceraldehyde-3-phosphate dehydrogenase (GapDH), fructose-1,6-bisphosphatase (Fbp1), phosphoenolpyruvate carboxykinase (Pck1), translation machinery (translation elongation factor 1α [Tef1], ribosomal protein L1b [RPL1b], alanyl-tRNA synthetase [Ars1]), heat shock protein 70 (Hsp70), superoxide dismutase (Sod1), and the cytoskeletal protein actin (Act1). Each column represents protein abundance in lysates from biological replicate yeast cultures (*n* = 4).

To identify the proteins comprising the *Histoplasma* yeast peroxisomal proteome, proteins biotinylated by peroxisome-localized TurboID (mNG:TurboID:PTS1, hereafter referred to as TurboID:PTS1) were compared to the proteins biotinylated by cytosol-localized TurboID (mNG:TurboID, hereafter referred to as TurboID). Four biological replicate cultures of *Histoplasma* TurboID/TurboID:PTS1-expressing yeasts were labeled by addition of 1 mM biotin for 6 h. Detection of biotinylated proteins from yeast expressing cytosolic or peroxisome-localized TurboID showed visible differences in the biotinylated protein profiles (Fig. 3A), confirming compartment-specific protein tagging with biotin. Biotinylated proteins from cellular lysates were captured with streptavidin-conjugated agarose resin, digested with trypsin, and identified via LC-MS/MS by matching peptide spectra to a database of translated genes encoded in the *Histoplasma* genome. Protein identities required representation by at least two unique peptides and detection in at least two of the four biological replicates. To normalize protein levels between replicates, we used the average abundance of the endogenously biotinylated proteins methylcrotonyl-CoA carboxylase (G217B_7030) and pyruvate carboxylase (G217B_5667) within each replicate (Table S1), since these proteins are captured independently of any biotinylation by TurboID. Principal component analysis of the normalized proteomic results confirmed that the identified TurboID-biotinylated proteins cluster strongly based on the compartment-specific localization of TurboID, with principal component 1 accounting for over 75% of the variance between data sets (Fig. 3B).

Comparative analysis of the proteomes revealed that 59 proteins were differentially enriched in the peroxisome (TurboID:PTS1) samples (at least twofold increased in peroxisome data sets and an adjusted *P*-value less than 0.05). Examination of the proteins which narrowly missed one of these cutoffs included a few proteins which are likely true peroxisomal matrix proteins, based on the presence of a predicted peroxisome-targeting sequence or verified peroxisomal localization in other organisms. Consequently, we adjusted the peroxisomal enrichment limit to include proteins which had a fold change of at least 1.8 and an adjusted *P*-value less than 0.1 (Fig. 3C). This revised analysis revealed 67 proteins differentially enriched in the peroxisome (TurboID:PTS1) samples (Fig. 3D; Table S2). Of these peroxisome-enriched proteins, 33 possessed a recognizable PTS1 based on the *Saccharomyces* consensus sequence [A/S/C/P]-[K/R/H]-L). The peroxisome-enriched proteins identified include several enzymes which carry out canonical functions of the peroxisomes, such as fatty acyl-CoA synthase and 3-ketoacyl-CoA thiolase (β-oxidation of fatty acids), isocitrate lyase and malate synthase (glyoxylate shunt), uricase (purine metabolism), and L-ornithine monooxygenase (siderophore biosynthesis) (48–53), the last of which has been validated as a peroxisome constituent in *Histoplasma* (18). In contrast, diverse proteins with known or predicted cytosolic functions (e.g., translation elongation factor 1α [RNA translation], fructose-1,6-bisphosphatase [gluconeogenesis], and actin [cytoskeleton]) were enriched in the cytosolic samples, as anticipated (Fig. 3E).

### Confirmation of peroxisomal localization of candidate peroxisomal proteins of *Histoplasma* yeasts

**Fig 4:**
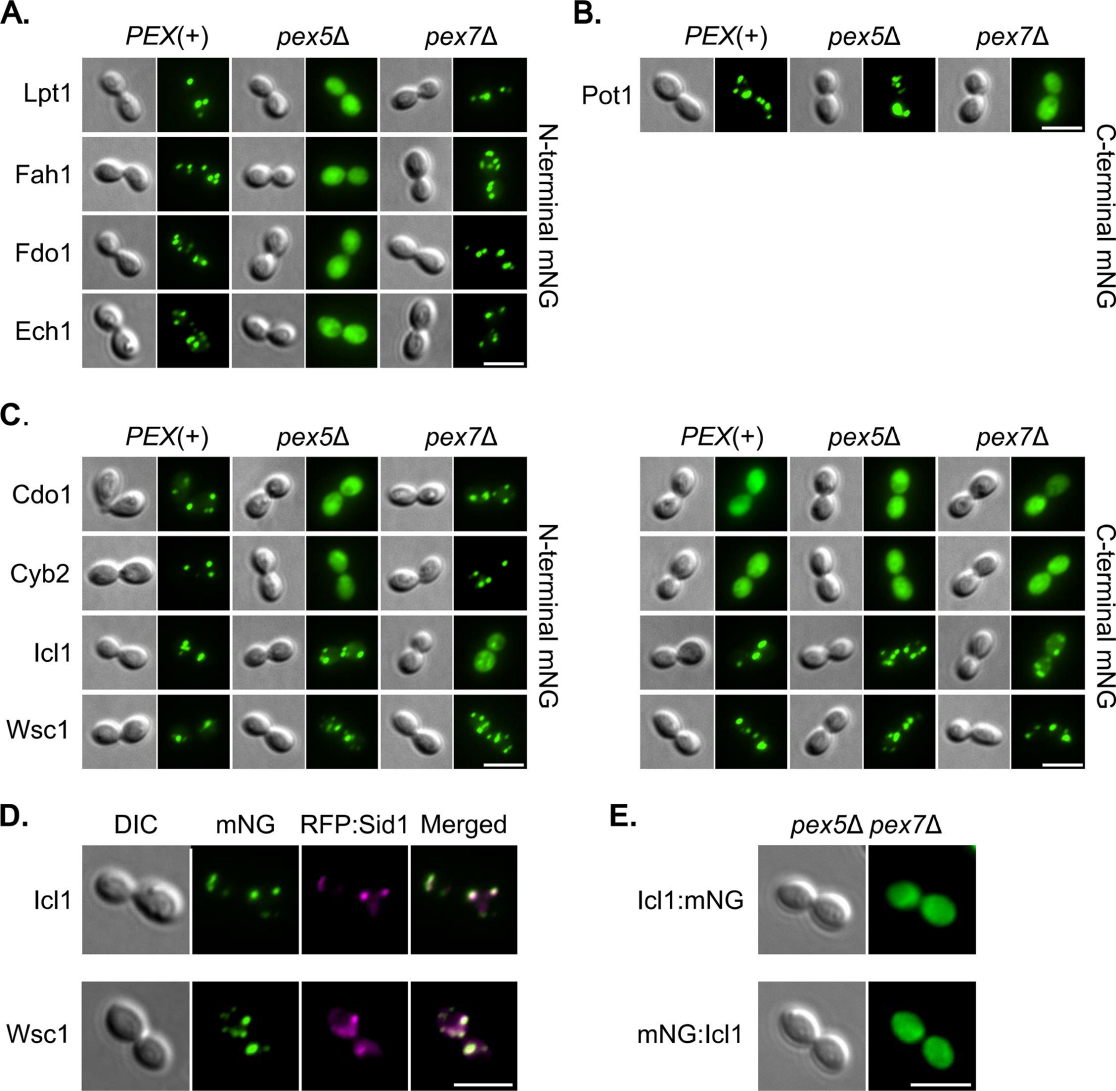
Localization of candidate peroxisomal proteins in *Histoplasma* yeasts. Differential interference contrast (DIC) and fluorescence microscopy images of candidate peroxisome-enriched proteins fused to N- or C-terminal mNG in *Histoplasma* with functional peroxisomal protein import [*PEX*(+)] or mutants lacking the PTS1- or PTS2 peroxisomal protein import pathways (*pex5*Δ or *pex7*Δ, respectively). Candidate proteins with predicted PTS1 (**A**) or PTS2 motifs (**B**) were fused with N-terminal mNG or C-terminal mNG, respectively, and expressed in *PEX*(+) *Histoplasma* yeasts or pex5Δ or pex7Δ mutant yeasts. (**C**) Proteins lacking identifiable PTS or PTS2 motif were fused to either N- or C-terminal mNG and the fusion proteins localized in *PEX*(+) *Histoplasma* yeasts or *pex5*Δ or *pex7*Δ mutant yeasts. (**D**) Verification of peroxisomal localization of the Pex5- and Pex7-independent proteins Icl1 and Wsc1. N-terminal mNG fusions to Icl1 and Wsc1 were expressed in *Histoplasma* yeasts expressing the peroxisomal protein Sid1 fused to a red fluorescent protein (RFP:Sid1) and the co-localization of proteins determined. (**E**) Localization of N- or C-terminal mNG:Icl1 fusion proteins in *pex5*Δ *pex7*Δ double mutant yeasts lacking both the PTS1 and PTS2 pathways. All panels show representative images of visualized yeasts (*n* > 50 yeasts analyzed). Scale bar represents 5 µm. Visualized proteins include Lpt1 (Lipid transfer protein 1, G217B_7495), Fah1 (Fumarylacetoacetate hydrolase, G217B_2697), Fdo1 (FAD-dependent oxidoreductase, G217B_6054), Ech1 (enoyl-CoA hydratase, G217B_5165), Pot1 (3-ketoacyl-CoA thiolase, G217B_8788), Cdo1 (Cysteine dioxygenase, G217B_7766), Cyb2 (L-lactate cytochrome c oxidoreductase, G217B_8374), Icl1 (Isocitrate lyase, G217B_1998), and Wsc1 (peroxisomal membrane protein, G217B_5352).

Although the presence of detectable PTS1 signals on some peroxisomal proteins suggests the legitimacy of the peroxisomal proteome, we confirmed peroxisomal localization of a subset of candidate peroxisomal proteins via microscopy to provide independent validation. In the absence of antibodies, fusions to mNG were constructed to monitor peroxisomal protein localization. The candidate proteins selected for localization represent diverse functions and biochemical characteristics. For proteins with a recognizable PTS1-targeting sequence (e.g., Lpt1 [lipid transfer protein], Fah1 [fumarylacetoacetate hydrolase], Fdo1 [FAD-dependent oxidoreductase], and Ech1 [enoyl-CoA hydratase]), mNG was fused to the N-terminus (Fig. 4A) to preserve the native PTS1 tripeptide at the C-terminus. For the peroxisome-enriched protein with a predicted PTS2-targeting sequence (Pot1 [3-ketoacyl-CoA thiolase]), which is near the N-terminus of the protein (54), mNG was fused to the C-terminus of the protein (Fig. 4B). For candidate proteins lacking predicted PTS signals (Cdo1 [cysteine dioxygenase], Cyb2 [L-lactate cytochrome c oxidoreductase], Icl1 [isocitrate lyase], and Wsc1 [Woronin sorting complex protein]), both N- and C-terminal mNG fusions were expressed (Fig. 4C). This sampling of peroxisomal proteins also includes one protein with predicted transmembrane regions (Wsc1) and one protein (Cdo1) which was included in the peroxisomal proteome due to the relaxed enrichment cutoffs. In addition, we tested the peroxisomal import pathway used for peroxisomal import of candidate proteins by expressing the mNG-protein fusions in yeasts lacking the PTS1- or the PTS2-import pathways. To facilitate these tests, deletions of the *PEX5* gene (responsible for import of PTS1-peroxisomal proteins) or the *PEX7* gene (responsible for import of PTS2-peroxisomal proteins) were generated using CRISPR/Cas9 methodology (55). The mNG-fusions proteins were expressed in *pex5*Δ or *pex7*Δ mutant yeasts as well as yeasts with intact *PEX*-dependent peroxisomal import functions (*PEX*(+) yeasts) (Fig. 4A through C).

For each candidate protein, the mNG-fusion construct displayed a punctate fluorescence pattern in wild-type [*PEX*(+)] *Histoplasma* yeasts indicating localization to the peroxisome. As expected, candidate peroxisomal proteins that contained a putative PTS1 sequence were mislocalized to the cytosol in yeasts lacking Pex5 function but remained unaffected by loss of Pex7 (Fig. 4A), confirming them as peroxisomal proteins that are imported via the PTS1 pathway. The opposite result was seen with the PTS2-containing Pot1 protein, which required Pex7 and not Pex5 for peroxisomal import (Fig. 4B), indicating Pot1 import via the PTS2 peroxisomal import pathway. For the Cdo1 and Cyb2 proteins that lack recognizable PTS1 or PTS2 sequences, loss of Pex5 or mNG placement at the C-terminus abolished their punctate localization (Fig. 4C), indicating their import into peroxisomes via the PTS1 pathway. Isocitrate lyase (Icl1) also lacks a recognizable PTS1 or PTS2 sequence but localizes to subcellular puncta (Fig. 4C). Interestingly, the loss of either Pex5 or Pex7 only partially prevented the localization of Icl1 to peroxisomes, with much of the fluorescently tagged Icl1 remaining in visible puncta. Like Icl1, Wsc1 localized to puncta independent of Pex5 and Pex7 (Fig. 4C), consistent with its identity as a membrane protein that is not imported into the peroxisomal matrix by Pex5 or Pex7, but instead inserted into the peroxisome membrane by the PMP import pathway (23). We confirmed that the fluorescent puncta observed for Icl1 and Wsc1 represented peroxisomes by colocalization of the mNG-tagged proteins with the established peroxisomal protein Sid1 (Fig. 4D). The retention of peroxisomal import of Icl1 in the absence of PTS1 and PTS2 import pathways suggests that Icl1 has redundant PTS1 and PTS2 signals or the operation of a third peroxisomal import mechanism. The peroxisomal import of Icl1 was abolished in a *pex5*Δ *pex7*Δ double mutant strain, which lacked both PTS import pathways (Fig. 4E), confirming that *Histoplasma* Icl1 is imported into peroxisomes through either the PTS1 or PTS2 pathway. The confirmed localization of all of these candidate peroxisomal proteins to the organelle provides strong evidence that the comparative proximity labeling approach defined true peroxisomal proteins.

### Comparative proteomics in peroxisomal import pathway mutants define Pex5- and Pex7-dependent peroxisomal proteins in *Histoplasma*

**Fig 5:**
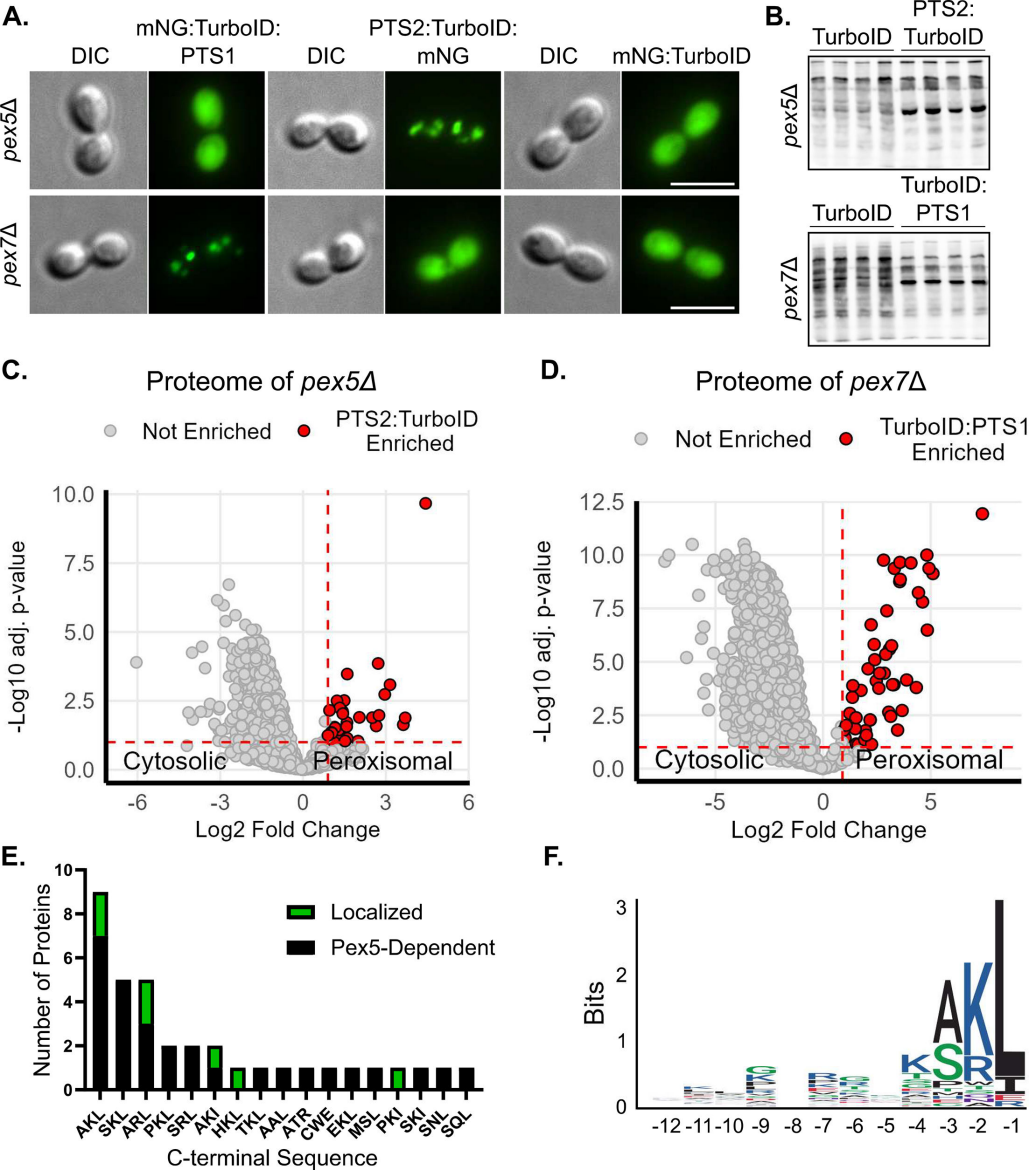
Proximity labeling of proteins in *Histoplasma* peroxisomal import mutants reveals PTS1- and PTS2-specific peroxisomal matrix proteins. (**A**) Pex5- and Pex7-dependent peroxisomal localization of TurboID harboring PTS1- or PTS2-signals. N-terminal mNG fusions to TurboID (mNG:TurboID) or TurboID with PTS1 (mNG:TurboID:PTS1) and C-terminal mNG fusion to TurboID with PTS2 (PTS2:TurboID:mNG) were expressed in Pex5- and Pex7-deficient yeasts (*pex5*Δ and *pex7*Δ, respectively), and the mNG localization was determined. (**B**) Biotinylated proteins from peroxisome-targeted TurboID (either TurboID:PTS1 or PTS2:TurboID)-expressing yeasts (lacking Pex5 or Pex7) were detected by HRP-conjugated streptavidin. (**C and D**) Volcano plots of peroxisomal-enriched biotinylated proteins in the *pex5*Δ (**C**) or *pex7*Δ (**D**) mutant strains. Peroxisome enrichment (red data points; adjusted *P*-value < 0.1 and fold enrichment > 1.866 in peroxisome-localized TurboID samples) of biotinylated proteins was determined by capture from yeasts expressing peroxisomal TurboID (either TurboID:PTS1 or PTS2:TurboID) compared to cytosolic TurboID. (**E**) Composition of C-terminal PTS1 signals among *Histoplasma* Pex5-dependent peroxisomal proteins. The C-terminal tripeptides of candidate PTS1-containing (Pex5-dependent import) peroxisomal proteins are listed (*x*-axis) with their frequency (*y*-axis) among the Pex5-dependent peroxisomal import protein population. Proteins verified by mNG-fusions to localize to the peroxisome in *Histoplasma* are indicated in green. (**F**) *Histoplasma* consensus PTS1 motif. Sequence logo shows the occurrence frequency of amino acids in the 12 C-terminal positions of the Pex5-dependent peroxisomal proteins in *Histoplasma*.

Our previous work discovered multiple roles for *Histoplasma* peroxisomes during infection, with distinct phenotypes related to the PTS1 and PTS2 pathways (18). Consequently, we sought to delineate which peroxisomal proteins were imported via PTS1-Pex5 or PTS2-Pex7 pathways by defining the Pex5- and Pex7-dependent peroxisomal proteomes. To accomplish this without reliance on bioinformatic predictions, we utilized a peroxisome-targeted TurboID to perform similar comparative proteomic analyses in yeasts lacking Pex5 or Pex7 function. As the previously utilized peroxisomal TurboID was imported via the PTS1 pathway and does not localize to the peroxisome in a strain lacking Pex5 function, we created a TurboID construct that was instead directed to the peroxisome via the PTS2 pathway. We fused the N-terminal 40 amino acids of the peroxisomal Pot1 protein, which include the predicted PTS2 sequence (RLSQVTSHL), to the N-terminus of TurboID:mNG. As expected, the PTS1-containing fluorescent TurboID fusions localized to peroxisomes in the Pex7*-*deficient but not the Pex5-deficient yeasts, and conversely, TurboID with the PTS2-targeting motif localized to peroxisomes in the Pex5-deficient but not in the Pex7-deficient yeasts (Fig. 5A). Expression of the two peroxisome-targeted TurboID proteins in yeasts lacking one of these import pathways, along with the addition of exogenous biotin, caused biotinylation of diverse proteins with 1D electrophoresis profiles that qualitatively differed from each other as well as from that of a cytosol-localized TurboID (Fig. 5B), indicating labeling of different peroxisomal proteins in the Pex5- and Pex7-deficient yeasts.

Comparative proteomics of lysates from peroxisome- and cytosol-localized TurboID in each *PEX*-deficient background empirically defined peroxisomal proteins imported by the PTS1 and PTS2 pathways. Comparison of proteomes from Pex5-deficient yeasts identified 33 proteins enriched in lysates from yeasts expressing the peroxisome-localized TurboID (PTS2:TurboID) (Fig. 5C), while comparison of proteomes from Pex7-deficient yeasts identified 50 proteins enriched in lysates from yeasts expressing the peroxisome-localized TurboID (TurboID:PTS1) (Fig. 5D). While many of these peroxisome-enriched proteins represented a subset of the total peroxisomal proteome previously defined in the *PEX*(+) strain (Fig. 3C), a portion, particularly in the *pex5*Δ background, represented newly identified proteins, possibly due to decreased peroxisomal matrix complexity due to a reduced number of peroxisomal proteins in the peroxisome *pex5*Δ mutant permitting greater sensitivity. To ensure stringency when identifying candidate peroxisomal proteins across these different backgrounds, we included an additional filtering step for these data sets: all proteins enriched in peroxisomes from the *PEX*-deficient backgrounds were cross-referenced to our *PEX*(+) peroxisomal proteome data set and removed if they did not show any level of peroxisome enrichment in our original experiment (i.e., log_2_ fold change for peroxisome enrichment < 0) (Table S3). This additional filtering step yielded a total of 40 candidate peroxisomal proteins from the Pex7-deficient data set and six candidate peroxisomal proteins from the Pex5-deficient data set. Importantly, three of the proteins identified in the Pex5-deficient data set (Pot1, Wsc1, and Icl1) were experimentally validated to traffic to the peroxisome without Pex5 function (Fig. 4). This data is consistent with findings in other organisms that more peroxisomal matrix proteins are imported via the PTS1 than the PTS2 pathway (21). Only three proteins were commonly enriched in peroxisomes in both the Pex5- and Pex7-deficient yeasts: two were membrane-bound proteins, which include the previously identified Wsc1 (Fig. 4C), and the Pex13 peroxin, which forms a critical part of the pore complex through which peroxisomal matrix proteins are shuttled into the organelle lumen. The only non-membrane bound protein which was significantly enriched in the peroxisome in all data sets was Icl1, consistent with its peroxisomal import by either Pex5 or Pex7 (Fig. 4C).

### Reassessment of the PTS1 consensus sequence in *Histoplasma*

After generating a more comprehensive set of proteins that localize to the peroxisome along with data on the relevant import pathways for each protein, we re-examined the PTS1 consensus sequence for *Histoplasma*. Thirty-four candidate peroxisomal proteins identified in this study were enriched in the peroxisome in each condition tested except in the strain lacking Pex5 function, assigning these proteins as peroxisomal matrix proteins imported by the PTS1-Pex5 pathway. Frequency analysis of the C-terminal three amino acids of each Pex5-dependent peroxisomal protein revealed a strong preference for -AKL, -SKL, or -ARL as these were the only PTS sequences which appeared in more than two proteins in the data set (Fig. 5E) (22). Among C-terminal sequences represented by only single Pex5-dependent matrix proteins, multiple tripeptide sequences (-AKI, -HKL, -PKL) were confirmed to sufficiently direct protein localization to the peroxisome (Fig. 4C and 5E), highlighting that rare tripeptides can still drive peroxisomal import. Fusion of mNG to additional tripeptides (-AKI, -HKL, and -PKL) led to peroxisomal localization of the fluorescent protein confirming that these atypical PTS1 signals are sufficient to drive PTS1 transport (Fig. S1). Amino acid compositional frequency analysis of the C-terminus of all the putative PTS1 sequences yielded a more relaxed consensus motif in *Histoplasma* (Fig. 5F) with the most frequent sequences still adhering to the *Saccharomyces* consensus. This analysis did not reveal any particular preference for certain amino acids preceding the C-terminal tripeptide.

**Fig 6:**
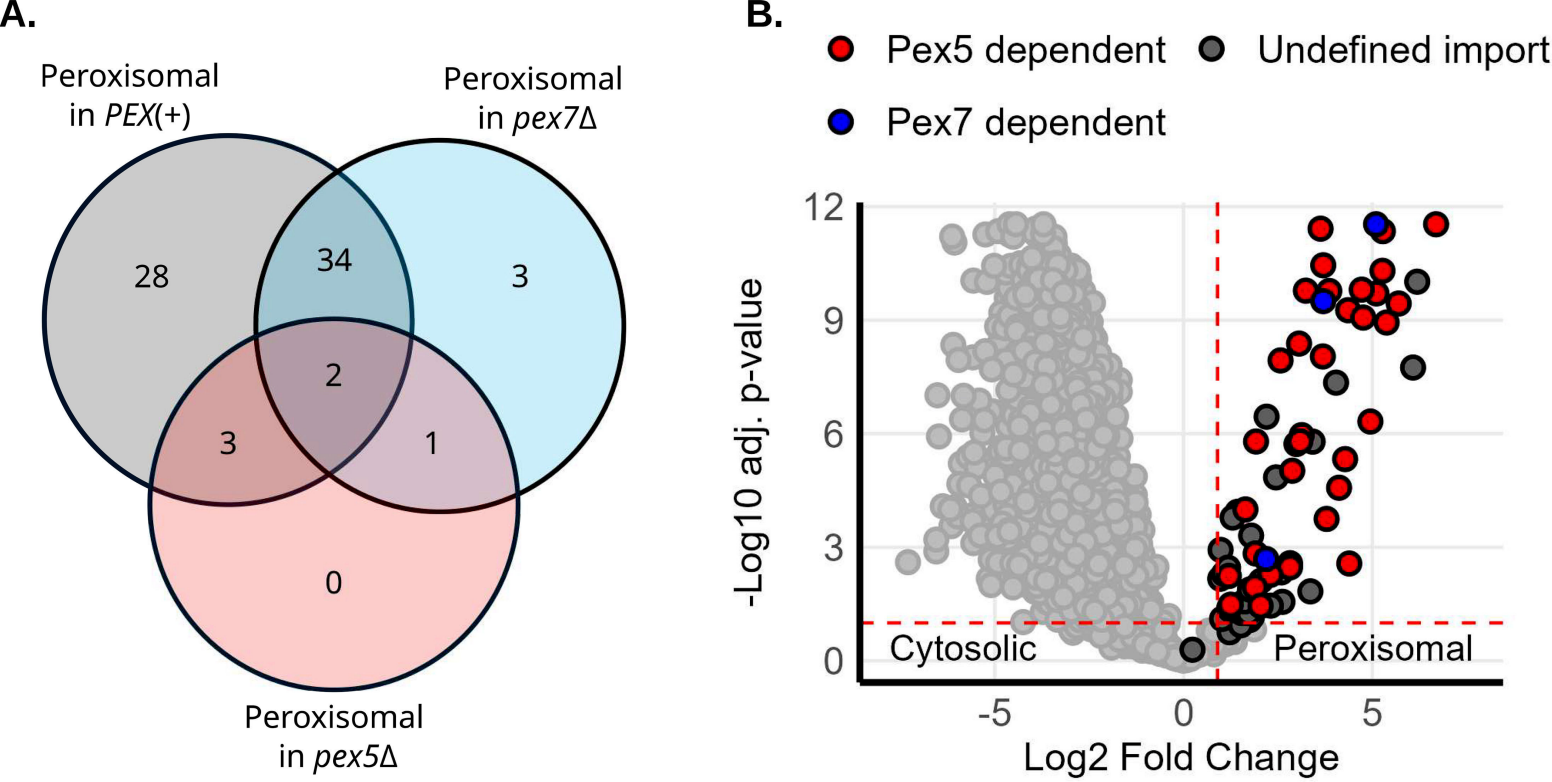
PTS pathway-dependent peroxisomal proteomes of *Histoplasma in vitro grown* yeast. *(***A**) Overlap of *Histoplasma* peroxisomal proteomes of *Histoplasma* PTS pathway-specific data sets of yeast from culture of yeasts in liquid media. The Venn diagram depicts the commonalities among peroxisomal proteomes of *PEX*(+), Pex5-deficient (*pex5*Δ*),* and Pex7-deficient (*pex7*Δ) strains, revealing peroxisomal proteins whose import is Pex5-dependent or Pex7-dependent. (**B**) Summary of the Pex5- and Pex7-dependent proteins imported into peroxisomes in the compiled total peroxisomal proteome. Proteins identified in *PEX*(+) and either the *pex5*Δ or *pex7*Δ data sets are designated as Pex5-dependent (red data points) or Pex7-dependent (blue data points), respectively, for peroxisomal import. Peroxisomal proteins identified in a single data set or lacking Pex5- or Pex7-dependent peroxisome enrichment are designated as undefined import. Fold change values and adj. *P*-values used for plotting were derived from the *PEX*(+) biological replicates.

Combining these data sets yields a peroxisomal proteome for *Histoplasma* yeast grown in media, consisting of 71 proteins. The vast majority of these proteins (67/71) were identified through comparison of proximity labeling data sets between cytosolic and peroxisomal TurboID in yeasts with intact peroxisomal import capacity [*PEX*(+)]. Four proteins were added based on proteomes from experiments with either Pex5- or Pex7-deficient yeasts (Fig. 6A). Of this aggregated proteome, 34 proteins are dependent on Pex5 for import into the peroxisome, and three proteins were no longer enriched in our analysis in the mutant strain lacking the Pex7 protein (Fig. 6B). One of these three identified proteins was Pex7 itself, which is not a peroxisomal matrix protein but is likely biotinylated while trafficking the PTS2:TurboID construct to the peroxisome. The remaining 34 identified peroxisomal proteins could not be strictly assigned to an import pathway based on the proteomes from Pex5- and Pex7-deficient yeasts.

### Determination of the peroxisomal proteome of intracellular pathogenic-phase *Histoplasma*

To more directly examine *Histoplasma* peroxisomes in the context of pathogenesis, we leveraged these proximity labeling techniques to characterize the peroxisomal proteome of *Histoplasma* yeasts during growth in macrophages. Previous work demonstrated that wild-type *Histoplasma* yeasts proliferate within these macrophages, but *Histoplasma* yeasts lacking peroxisomal import are deficient for intracellular growth (18), confirming that *Histoplasma* peroxisome functions are active and required during macrophage infection. To generate peroxisomal protein samples, *Histoplasma* yeast expressing either TurboID or TurboID:PTS1 were used to infect cultured macrophages. After 18 h, addition of biotin to the culture for 6 h initiated biotin labeling of proteins in the yeast cytosolic or peroxisome compartments. Following labeling, yeasts were recovered from the macrophages and the yeasts lysed to capture the biotinylated proteins. We modified our workflow in this experiment to allow for additional recovery and identification of peroxisomal membrane proteins by adding 1% Tween-20 to the yeast lysates.

**Fig 7:**
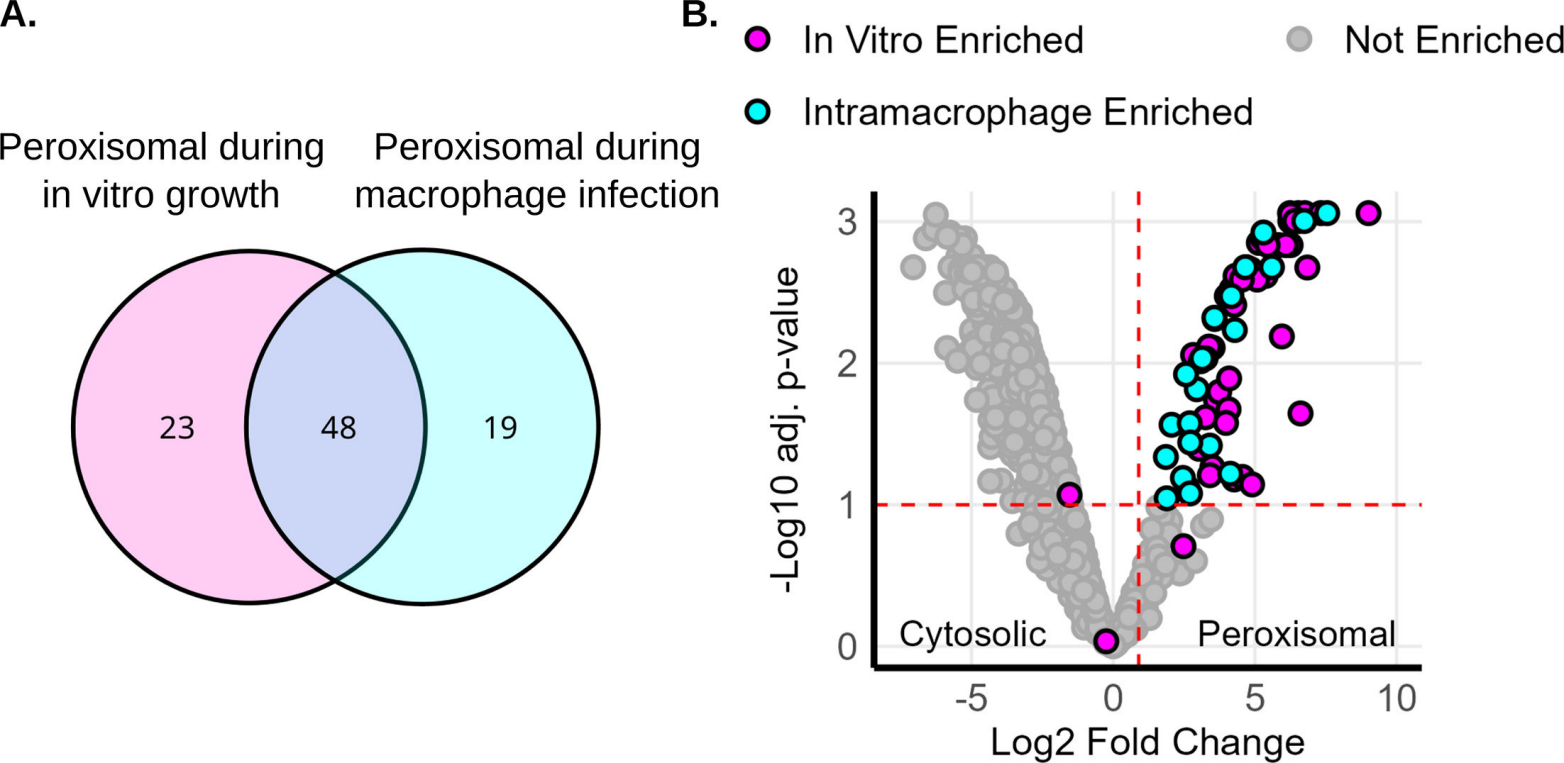
Determination of the *Histoplasma* peroxisomal proteome during macrophage infection. (**A**) Comparison of the aggregated peroxisomal proteomes of *Histoplasma* identified during yeast-phase growth with peroxisome-enriched proteins identified via proximity labeling of *Histoplasma* yeasts during infection of P388D macrophages. Venn diagram depicts the overlap of *Histoplasma* peroxisomal proteins identified from yeasts grown in media compared to peroxisomal proteins identified for yeasts recovered from macrophages after 24 h of infection. (**B**) Summary of the *Histoplasma* peroxisomal proteins identified during macrophage infection and concordance with *in vitro* yeast culture data sets. Fold change and adj. *P*-values used for plotting were derived from the intramacrophage data set. Graph plots proteins enriched in intracellular TurboID:PTS1-expressing yeasts (peroxisomal) compared to intracellular TurboID-expressing yeasts (cytosolic) (*x*-axis). Data point colors indicate peroxisome-enriched proteins also identified *in vitro* grown yeasts (magenta), proteins specific for the yeasts recovered from macrophages (cyan), and proteins that showed no peroxisome enrichment in any data set (gray).

Comparative proteomic analysis of the TurboID and TurboID:PTS1-expressing yeasts from macrophages revealed 67 proteins that were significantly enriched in the peroxisome (i.e., samples from TurboID:PTS1-expressing yeasts) compared to the cytosol (samples from TurboID-expressing yeasts) (Table S4). These newly identified proteins displayed high concordance with the peroxisome profiling of yeasts in broth culture, as 48 of the 67 yeast peroxisomal proteins were enriched in both *in vitro* (broth culture) and infection conditions (Fig. 7A). The 19 enriched proteins which were newly identified during macrophage infection included seven proteins which contain a predicted PTS1 sequence and multiple membrane-bound proteins, including the peroxisomal ATP transporter Ant1 as well as a key member of the peroxin pore complex, Pex14. The new proteins also included the siderophore biosynthesis protein Sid3 which has previously been identified as a peroxisomal matrix protein in *Histoplasma* (18)*,* confirming peroxisome-dependent siderophore biosynthesis by intracellular yeasts. Three peroxisomal proteins defined from *in vitro* peroxisomal proteomes were no longer enriched in peroxisomes during infection (Fig. 7B) due to lower fold-enrichment (G217B_8342, enrichment of 0.34 and G217B_5270, enrichment of 0.84) or high variability (G217B_7708, *P* adj. = 0.19). Combined, these data sets obtained from *Histoplasma* yeasts from multiple growth conditions (broth culture and infection) yielded a high-confidence peroxisomal proteome of pathogenic-phase *Histoplasma*, consisting of 90 proteins that were enriched in at least one experiment (Table S5) and highlight peroxisome functions operating in pathogenic-phase *Histoplasma* during macrophage infection.

## DISCUSSION

Peroxisomes play multiple roles in the virulence of *Histoplasma,* and the required functions are dependent on both the PTS1 and PTS2 peroxisomal import pathways (18). Our previous work demonstrated that siderophore synthesis requires the peroxisomal matrix protein Sid1 similar to other fungi (31). Interestingly, *Histoplasma* siderophores are only required following activation of cell-mediated immunity (18). However, additional pathogenesis-enabling functions of the peroxisome are distinguishable from siderophore biosynthesis since peroxisomal matrix import is also required for pathogenesis before activation of cell-mediated immunity. These earlier peroxisome functions depend on Pex5, indicating that critical peroxisomal matrix proteins are imported via the PTS1 import pathway. Thus, definition of the *Histoplasma* peroxisomal proteome, and PTS1-containing proteins in particular, provides the foundation for continued study of the role of peroxisomes in *Histoplasma* pathogenesis.

TurboID-based proximity labeling of proteins within the peroxisome and comparative proteomics identified 90 constituent proteins of *Histoplasma* peroxisomes. These proteins likely represent the majority of the peroxisomal matrix proteins in pathogenic-phase *Histoplasma* cells given the repeated identification of many of these proteins in multiple data sets. Furthermore, identification of 90 proteins is comparable to characterizations of other peroxisomes using independent methodologies. For example, organellar proteomics revealed 79 predicted peroxisomal matrix proteins in *Penicillium chrysogenum* (33), and purification of glycosomes from *Neurospora crassa* identified 191 proteins, of which only 38 were considered likely peroxisomal proteins based on PTS predictions and likely peroxisome function (56). Cell fractionation combined with isotope-coded affinity tag (ICAT) techniques identified 70 proteins in *Saccharomyces cerevisiae* (34). An orthogonal approach to proteomics using microscopy to localize fluorescent fusion proteins in *Saccharomyces cerevisiae* identified 33 additional peroxisomal proteins, bringing the total number of peroxisome-associated proteins in *S. cerevisiae* to 115 (36). It is important to note that this increased number represents proteins that were discovered via multiple techniques and multiple growth conditions as well as the peroxins in that organism. We acknowledge additional proteins could be found in the peroxisome of avirulent mycelial-stage *Histoplasma* cells; however, given our focus on the virulence-enabling functions of the *Histoplasma* peroxisome, we focused on definition of the peroxisomal proteome from pathogenic-phase *Histoplasma* yeasts.

The definition of the *Histoplasma* peroxisome during intracellular growth in macrophages provides a more relevant link between the peroxisomal proteome and pathogenicity. Over 70% of the identified peroxisomal proteins were shared between peroxisomal proteome data sets from *in vitro* culture or macrophage infection, suggesting that expression of many of the peroxisome functions is constitutive or regulated by differentiation into pathogenic yeasts. Of the 23 peroxisomal proteins identified from yeasts grown in media but not in intracellular yeast, 20 proteins were not identified from yeasts during infection and three were below significant peroxisome enrichment. Although this may indicate downregulation of some proteins during infection, it is likely that this resulted from reduced sensitivity of the samples from intracellular yeasts due to scaling challenges for determination of the peroxisomal proteome following infection. Technical challenges (feasible number of host macrophages that could be cultured and imperfect recovery of yeasts from macrophages) resulted in reduced intracellular yeasts, amounting to 10% of the yeasts profiled for *in vitro* growth, and consequently reduced the depth of proteome samples. Nonetheless, macrophage infection still resulted in the identification of additional peroxisomal proteins, possibly representing peroxisome functions induced by the infection environment, including additional ROS-detoxification enzymes such as a superoxide dismutase (G217B_8545) and peroxiredoxin (G217B_8481).

The 90 peroxisomal proteins identified from *Histoplasma* yeasts are high-confidence constituents of the peroxisome, but the proteome should not be considered as an exhaustive proteome. Our experiments did not identify *Histoplasma’s* CatP peroxisomal catalase which is known to localize to peroxisomes (18), likely because the PTS1 of CatP (-PRL) causes only partial peroxisomal localization of the catalase in *Histoplasma* (Fig. 2B). Consistent with this, CatP was found in both cytosolic and peroxisome samples, yielding an enrichment of only 0.61-fold, which was below our threshold. This -PRL targeting sequence is absent in all other identified proteins dependent on Pex5 for peroxisomal import, suggesting that -PRL may not drive efficient targeting of peroxisomal proteins. Such incomplete peroxisomal import of the peroxisomal catalase also characterizes the *Ogataea polymorpha* catalase which also has an uncommon PTS1 sequence (-SKI). Replacement of this tripeptide with a conventional -SKL sequence causes more complete peroxisomal import (57). This example shows that this comparative methodology can miss dual-localized proteins (cytosolic and peroxisomal) as peroxisome constituents. In addition, our peroxisomal proteome captured a limited number of peroxisome membrane proteins resulting in only partial characterization of the peroxisomal membrane. Inclusion of detergents during sample processing appeared to increase the recovery of several hallmark peroxisomal membrane proteins such as Ant1 and Pex14. Nonetheless, the identified total peroxisomal proteome in this study defines peroxisomal matrix proteins whose import is mediated by Pex5 and that represent candidate PTS1-containing proteins linked to *Histoplasma* pathogenesis.

Rather than rely on bioinformatic predictions, the experimentally derived peroxisomal proteome identified PTS1-containing peroxisomal proteins by extending comparisons to a Pex5-deficient *Histoplasma* strain. This direct approach defined 34 *Histoplasma* proteins as matrix constituents that depend specifically on the PTS1-Pex5 import pathway. Consistent with studies in other fungi, most proteins in the peroxisomal proteome are PTS1/Pex5-dependent. While much of the PTS1 import machinery is conserved across eukaryotes, the same PTS1 tripeptide may be sufficient for localization in one fungus but not in another (58). Indeed, we observed some divergence from the *Saccharomyces* consensus PTS1 sequence, with the *Histoplasma* peroxisomal proteins dependent on Pex5 for import containing a revised C-terminal motif of [A/S/P/H/C][K/R][L/I]. This motif includes amino acids in all instances where they appear at least twice in that position among our Pex5-dependent proteins, or at least once in the tripeptide of a protein that has verified Pex5-dependent localization via microscopy with a fluorescent fusion protein. This suggests that more accurate bioinformatic predictions of peroxisome-localized proteins may first require an analysis of experimentally defined PTS1-type matrix proteins. However, alternative targeting mechanisms that are Pex5-dependent but not reliant on a C-terminal tripeptide could complicate consensus definition. This includes proteins potentially imported to peroxisomes via “piggybacking” with another Pex5-dependent peroxisomal protein (59, 60). Another example of alternative Pex5-dependent import involves formation of a “PTS3” region, in which several distant amino acids are brought into close proximity in the folded protein conformation and form a signal patch that binds Pex5 without a C-terminal PTS1 (61). Nonetheless, the identification of 34 Pex5-dependent peroxisomal matrix proteins in *Histoplasma* in this experiment, whether or not they adhere to a PTS1 consensus, provides the foundation for investigating which proteins contribute to *Histoplasma* virulence, as yeasts lacking Pex5 are significantly attenuated during infection (18).

Examination of the predicted protein functions represented in the peroxisomal proteome reveals many well-documented functions of peroxisomes, as well as several potentially novel functions of the organelle. The *Histoplasma* pathogenic-phase peroxisomal proteome includes β-oxidation enzymes (fatty acyl-CoA synthase Faa1 [G217B_3886], fatty acyl-CoA oxidase Pox1 [G217B_2043], 3-ketoacyl-CoA thiolase Pot1 [G217B_8788], and Δ3, Δ2-enoyl-CoA isomerase Eci1 [G217B_7642]) and enzymes of the glyoxylate shunt (isocitrate lyase Icl1 [G217B_1998] and malate synthase Mls1 [G217B_7708]). In addition, the *Histoplasma* peroxisomal proteome contains the siderophore proteins Sid1 (G217B_4779) and Sid3 (G217B_4777), as well as a D-amino acid oxidase (G217B_0638).

Several proteins of the *Histoplasma* peroxisomal proteome suggest metabolic functions not typically associated with peroxisomes, including diverse metabolisms of amino acids. These functions are intriguing as studies point to amino acids as critical sources of carbon, sulfur, and potentially nitrogen to support *Histoplasma* intracellular growth during infection (13, 15, 62). For example, the *Histoplasma* peroxisome contains a predicted fumarylacetoacetate hydrolase (Fah1, G217B_2697), which performs the final step in the catabolism of tyrosine and phenylalanine and is essential for incorporation of tyrosine- and phenylalanine-derived carbon into the TCA cycle (63). Tyrosine and phenylalanine are both potentially relevant metabolites for pathogenic-phase *Histoplasma*, as aromatic amino acid catabolism enzymes are upregulated in yeast grown on amino acids, intracellular yeasts are characterized by elevated phenylalanine (62), and tyrosine and phenylalanine are sufficiently available in the macrophage phagosome to rescue the growth of tyrosine and phenylalanine auxotrophs (64). The *Histoplasma* peroxisomal proteome characterized in media and intramacrophage settings also includes a cysteine dioxygenase Cdo1 (G217B_7766), which contributes to intracellular cysteine homeostasis. In *Candida albicans,* the homologous cysteine dioxygenase, Cdg1, converts excess cysteine to sulfate for subsequent excretion to eliminate cysteine toxicity (65). Given that cysteine is a potential source of organic sulfur for intracellular yeasts (62), the peroxisome-localized Cdo1 is an intriguing candidate for how peroxisomes might contribute to pathogenesis. The data set collected from intramacrophage yeast specifically identified the predicted 3-hydroxyisobutyryl (HIBYL)-CoA hydrolase (G217B_5424) as a peroxisomal constituent. Proteins in this family have been linked to valine metabolism in many organisms (66, 67). HIBYL-CoA hydrolase plays a critical role in this context by regulating the equilibrium of 3-hydroxyisobutyryl-CoA to prevent upstream accumulation of the toxic methacrylyl-CoA intermediate (68). In *Arabidopsis*, the peroxisomal Chy1 HIBYL-CoA hydrolase protein is associated with branched-chain amino acid degradation and metabolism of propionate and isobutyrate, likely through a modified β-oxidation pathway as seen in *Candida albicans* (69–72). These multiple links to amino acid metabolism functions in the peroxisome are intriguing, given the potential of amino acids as a nutritional source for *Histoplasma* to proliferate within the phagosome of macrophages.

Definition of the peroxisomal proteome through proximity labeling has several strengths compared to organelle isolation or bioinformatic predictions. Organelle isolation proves difficult for many organisms, particularly fungi such as *Histoplasma*, where disruption of the cell wall to release cellular contents is often frustrated by ineffectiveness or incompleteness of enzymatic cell wall degradation (data not shown). Even in model systems with well-optimized cellular fractionation techniques, contamination with other organelles, such as mitochondria, is common (73), resulting in the need to curate enriched proteins to a smaller, high-confidence list based on presence of a predicted PTS sequence (33, 56) or predicted functions. While this curation increases confidence, it prevents the identification of unsuspected proteins. In contrast, the subcellular localization of the TurboID proximity labeling enzyme provides confidence in the specificity of the resulting proteomics. The comparative approach using cytosolic- versus peroxisome-labeling narrows the proteome to a high-confidence set of peroxisomal proteins. The tractable nature of proximity labeling for the identification of organelle proteomes makes this methodology attractive for organisms with molecular genetic techniques that facilitate the expression of transgenes and their localization to specific subcellular compartments.

Proximity labeling using peroxisome-localized TurboID enabled us to define the peroxisomal proteome of pathogenic *Histoplasma* yeasts. The peroxisomal proteome includes matrix proteins that are expressed and imported into the organelle during *Histoplasma* infection of macrophages. Furthermore, our empirical, rather than bioinformatic, definition of matrix proteins dependent on Pex5 for peroxisomal import identifies candidate proteins and functions contributing to the virulence-associated role for the PTS1 pathway of peroxisomal import in this pathogen.

## MATERIALS AND METHODS

### Growth and maintenance of *Histoplasma capsulatum*

The *Histoplasma* strains used in this study (Table S6) are derived from the North American clade two clinical isolate G217B (ATCC 26032). For proteome derivations, *Histoplasma* yeasts were grown in *H. capsulatum*-macrophage medium (HMM) (74) or modified RPMI (RPM-1640 supplemented with 2% glucose, 50 mM glutamate, 10 mM HEPES, 50 nM CuSO_4_, 3 µM ZnSO_4_, and buffered to pH 5.5) at 37°C with continuous shaking until exponential phase growth. For maintenance of strains, strains were grown on HMM solidified with 0.6% agarose and supplemented with 25 µM FeSO_4_. For growth of uracil auxotrophs, media were supplemented with 100 µg/mL uracil. For selection of expression constructs with hygromycin-resistance, hygromycin B (50 µg/mL) was added to media.

### Generation of TurboID-expressing *Histoplasma* strains

For generation of TurboID-expressing strains in *Histoplasma*, the TurboID coding sequence from the V5-TurboID-NES_pCDNA3 plasmid (AddGene [40]) was employed. For expression of cytosolic TurboID, TurboID was amplified by PCR and cloned as a C-terminal fusion to monomeric NeonGreen in the expression vector pCR853, in which transgenes are constitutively expressed from the *TEF1* (translation elongation factor 1) promoter. For peroxisome-localized TurboID, the sequence encoding the C-terminal 20 amino acids (-VVNFGNVTSNGTAKPSKSKL) from Sid3 were added to the C-terminus of the mNG:TurboID. To generate a PTS2-localized TurboID, the sequence encoding the N-terminal 40 amino acids (-MSQAAQRLSQVTSHLTGKGGVSAITAKHPDDIVVTCALRT) from the PTS2-containing protein Pot1 (G217B_8788) was fused to the N-terminus of a TurboID:mNG construct. Expression plasmids were electroporated into *Agrobacterium tumefaciens* strain LBA1100 (75), which was subsequently used to transform *Histoplasma* strain WU15 [*PEX*(+)], OSU445 (*pex5*Δ), or OSU641 (*pex7*Δ), with selection for uracil prototrophy (76).

### Proximity labeling of subcellular compartments

For experiments performed on yeasts from broth culture, TurboID-expressing *Histoplasma* yeasts were cultured in modified RPMI at 37°C with continuous shaking until exponential phase (approximately 96 h). For proximity labeling 5 × 10^9^ cells were harvested and washed three times with sterile H_2_O before resuspension in 2 mL of modified RPMI with or without 1 mM biotin. The cultures were incubated for 6 h unless otherwise indicated, then collected, washed three times with sterile H_2_O, and resuspended in 800 µL phosphate-buffered saline (PBS; 137 mM NaCl, 2.7 mM KCl, 10.14 mM sodium phosphate, pH 7.4).

Cell lysates were generated by disrupting cells with 0.5 mm-diameter glass beads. Lysates were clarified by centrifugation at 3,000 × *g* for 5 min, followed by a second centrifugation at 16,000 × *g* for 10 min. 600 µL of supernatant was recovered for analysis, and the protein content was quantified using the BCA assay.

For labeling of cytosolic and peroxisomal proteins in intracellular yeasts, TurboID-expressing yeasts were used to infect 3 × 10^7^ P388D1 murine macrophages (77) in 150-mm-diameter plates at 37°C with 5% CO_2_/95% air in Ham’s F-12 media containing 10% fetal bovine serum. Macrophages were infected with TurboID-expressing *Histoplasma* at a multiplicity of infection of 4:1. After 18 h, 1 mM biotin was added to the media, and the infected cultures were incubated for an additional 6 h. Intracellular *Histoplasma* yeasts were recovered from macrophages by washing macrophages with PBS and then lysing the macrophages by adding 10 mL ice-cold water and scraping of the cells from the plate. To ensure efficient lysis, the macrophage lysate was passed through a 25 g needle, and the intracellular yeasts were collected by centrifugation at 4°C (5 min at 3,000 × *g*), washed twice with 5 mL ice-cold sterile water, and resuspended in 800 µL PBS. Yeast lysates were processed as described above with the addition of 1% Tween-20 to the lysate.

Fifteen micrograms of protein from clarified cell lysates was resolved via 1D SDS-PAGE and transferred to Protran 0.2 µm-pore NC nitrocellulose (Cytivia). Membranes were blocked in 4% (wt/vol) milk and washed three times with Tris-buffered saline (20 mM Tris, 145 mM NaCl, pH 7.5) containing 0.5% Tween-20. Membranes were probed with HRP-conjugated streptavidin (Thermo Scientific) diluted in Tris-buffered saline with 2% bovine serum albumin. Bound HRP-conjugated streptavidin was visualized by chemiluminescence using Radiance ECL HRP substrate (Azure Biosystems).

For capture of biotinylated proteins, clarified lysates were adjusted to 0.5% Tween-20 to limit non-specific protein binding. One hundred microliters of streptavidin-conjugated agarose (Pierce) was blocked in 2% BSA in PBS for 1 h, washed three times with PBS, and incubated with 500 µL of clarified lysates at room temperature with shaking for 1 h. For samples generated from the *pex5*Δ and *pex7*Δ mutants or from intramacrophage yeasts, 300 µL of streptavidin-conjugated beads were used and incubated with clarified cell lysates overnight. After biotinylated protein capture, the agarose resin was collected via centrifugation (10,000 × *g* for 2 min) and successively washed three times with 500 µL of 2% SDS, 8 M urea, 2 M NaCl, and 50 mM ammonium bicarbonate buffer (pH 7.8) before final resuspension in 200 µL of 50 mM ammonium bicarbonate buffer.

### Mass spectrometry-based identification of proteins

To prepare proteins for mass spectrometry, captured biotinylated proteins were reduced by the addition of dithiothreitol (DTT) to a concentration of 125 ng/µL, followed by incubation at 65°C for 15 min. Disulfide formation was blocked by the subsequent addition of iodoacetamide to a concentration of 375 ng/µL in the dark at room temperature for 30 min. Peptides were generated by the addition of 1 µg of sequencing grade-modified trypsin (Promega, Madison WI) in 50 mM ammonium bicarbonate buffer to the agarose resin at 37°C overnight. Trypsin digestion was quenched by the addition of 10 µL of 10% formic acid, and the peptides were separated from the resin by centrifugation (20,000 × *g* for 10 min). Peptide-containing supernatants were dried using a vacuum concentrator and were resuspended in 20 µL of 0.1% formic acid for LC-MS/MS analysis.

Peptides were identified by nano-liquid chromatography-nanospray tandem mass spectrometry (Nano-LC-MS/MS). Samples (6.4 µL) were injected into a µ-Precolumn Cartridge (Thermo Scientific) and desalted with 0.1% formic acid in water for 5 min. Samples were injected, and the peptides were separated on an Easy-Spray nano column (Pepmap RSLC, C18 3µ 100A, 75 µm × 150 mm; Thermo Scientific) using a 2D rapid separation liquid chromatography (RPLC) HPLC system (Thermo Scientific), with 0.1% formic acid in water as mobile phase A and acetonitrile (with 0.1% formic acid) as mobile phase B. Flow rate was set at 300 nL/min. Mobile phase B was increased from 2% to 16% for 105 min, 16–25% for 10 min, 25–85% for 1 min, and 95% for 4 min before being brought back to 2% for 1 min. The column was equilibrated at 2% of mobile phase B for 15 min prior to the next sample injection.

Tandem mass spectrometry (MS/MS) data were acquired using an Orbitrap Eclipse mass spectrometer (Thermo Scientific) equipped with a nanospray FAIMS Pro Source, operated in positive ion mode with a spray voltage of 1.95 kV and a capillary temperature of 305°C. The mass spectrometer scan sequence was based on the preview mode, data-dependent TopSpeed method: the analysis was programmed for a full scan recorded between *m*/*z* 375 and 1,500, followed by an MS/MS scan to generate product ion spectra for amino acid sequence determination, with consecutive scans targeting the most abundant peaks in the spectrum over the next 3 s. To achieve high mass accuracy MS determination, the full scan was performed in Fourier transform (FT) mode, and the resolution was set at 120,000 with internal mass calibration. Three compensation voltages (−40, −60, and −80v) were used for acquisition. The Automatic Gain Control (AGC) target ion number for FT full scan was set at 4 × 10^5^ ions, with a maximum ion injection time of 50 ms and one micro scan. Multi-stage mass spectrometry (MSn) was performed using higher-energy collisional dissociation (HCD) in ion trap mode to ensure the highest signal intensity of MSn spectra. The HCD collision energy was set at 28%. The AGC target ion number for ion trap MSn scan was set at 3.0 × 10^4^ ions, with a maximum ion injection at 35 ms and micro scan number at 1. Dynamic exclusion was enabled with a repeat count of one within 30 s and a low and high mass width of 10 ppm.

The resulting data were used to generate .mgf files, which were searched using Mascot Daemon (Matrix Science v 2.7.0) (78) against an in-house–generated *Histoplasma* genome encoded proteome using the Proteome Discoverer platform (Thermo Fisher Scientific). The in-house database was derived from an RNAseq-based manual curation of the *Histoplasma* genome (C. A. Rappleye, unpublished data). Mass accuracy of the precursor ions was set to 10 ppm, and inclusion of one ^13^C peak was allowed in the search. The fragment mass tolerance was set to 0.5 Da. Considered variable modifications included oxidation (Met), deamidation (N and Q), ubiquitylation (K), phosphorylation (S/T), and carbamidomethylation (Cys). Up to four missed trypsin cleavages in the identified peptides were permitted. A decoy database composed of the reversed peptide sequences from the genome-encoded proteome was also searched to determine the false discovery rate (FDR). The significance threshold was set at *P* < 0.05. Proteins with a significance of *P* < 0.05, an FDR of less than 1% FDR, and represented by a minimum of two peptides were considered valid.

### Comparative proteome analysis

Relative quantitation was performed using a label-free quantitation (LFQ) mass spectral peak intensity approach. Peptide precursor (MS1) intensities from the same protein were generated from Proteome Discoverer MASCOT search and summed for quantitation comparison. Prior to analysis, the protein intensities in each replicate sample were normalized to the average intensity of two endogenously biotinylated proteins in *Histoplasma* (pyruvate carboxylase, G217B_5667, and methyl-crotonyl-CoA carboxylase, G217B_7030). Normalized LFQ peak intensities were compared between samples using the DEP package (version 1.26.0) in R, and significance was assessed using Benjamini-Hochberg adjusted *P*-values. Proteins with adjusted *P*-values < 0.1 and log_2_ fold change values > 0.9 (>1.866 absolute fold change) in the peroxisome-targeted TurboID samples (mNG:TurboID:PTS1) compared to cytosolic TurboID (mNG:TurboID) were considered significantly enriched. A similar analysis was performed for samples from the *pex5*Δ and *pex7*Δ mutant strains (comparison of proteins from mNG:TurboID:PTS1 versus mNG:TurboID and PTS2:mNG:TurboID versus mNG:TurboID, respectively). Peroxisome-enriched proteins from the *pex5*Δ and *pex7*Δ experiments were retained only if they also showed increased abundance in the peroxisome-labeled samples compared with *PEX*(+) sample set (i.e., log_2_ fold change values > 0 in the comparison of mNG:TurboID:PTS1 vs mNG:TurboID).

Assignment of dependence on Pex5 or Pex7 for protein import into peroxisomes (representing the PTS1 and PTS2 pathways, respectively) was by comparison of the proteomes from the *pex5*Δ and *pex7*Δ mutants with the peroxisomal proteome from *PEX*(+) yeast cells: proteins significantly enriched in the peroxisomal proteome in the *PEX*(+) data set and the *pex7*Δ data set were classified as Pex5-dependent. Proteins significantly enriched in the peroxisomal proteome of the *PEX*(+) data set and the *pex5*Δ data set were classified as Pex7-dependent. Proteins that were only significantly enriched in a single data set or which were enriched in the peroxisomal proteome in all three genetic backgrounds were not assigned to a pathway.

### CRISPR-mediated generation of *Histoplasma* peroxisomal import mutants

Deletion of the *PEX5* or *PEX7* locus was performed by CRISPR/Cas9-mediated gene editing (55) using co-expression of two *PEX5*- or *PEX7*-targeting CRISPR guide RNAs (PEX5: AAGCCGAATAGAAGTACGCG and AGTTGGCGAGCGTAGCCCCG; PEX7: GACTTGACGTTGAACCACCG and GATGCGGGCTTCATGCATGG). Isolated mutants were sequenced which demonstrated a 1,747 bp deletion of the *PEX5* locus and a 1,320 bp deletion of the *PEX7* locus, removing 80% and 85% of the Pex5- and Pex7-coding sequences, respectively. The double *pex5*Δ *pex7*Δ mutant was generated by CRISPR-mediated deletion of the *PEX5* locus in the pex7Δ mutant.

### Localization of proteins to peroxisomes in *Histoplasma*

To generate fluorescent strains with different PTS1 tripeptides in *Histoplasma*, the mNG fluorescent protein was amplified by PCR with primer-encoded sequences for the PTS1 tripeptides from the known peroxisomal proteins CatP (-PRL), Sid1 (-ARL), or Sid3 (-SKL) at the C-terminus. These constructs were placed into the URA5-containing *Agrobacterium*-based T-DNA expression vector pCR853. Each of these constructs was transformed via *Agrobacterium*-mediated transformation into *Histoplasma* strain WU15, selecting for uracil prototrophy. *Histoplasma* candidate peroxisomal protein-encoding genes were amplified by PCR and fused to sequences for either an N-terminal or C-terminal mNG. These constructs were transformed via *Agrobacterium*-mediated transformation into *Histoplasma* strain WU15 [*PEX*(+)], OSU445 (*pex5*Δ), OSU641 (*pex7*Δ), or OSU737 (*pex5*Δ, *pex7*Δ), selecting uracil prototrophy (76). For colocalization with the Sid1 protein, a plasmid encoding hygromycin resistance and an *N*-terminal td-Tomato red-fluorescence protein (RFP) gene fusion to *SID1* (18) was transformed via *Agrobacterium-*mediated transformation into *Histoplasma* expressing mNG fusions to TurboID:PTS1, Icl1, and Wsc1. The mNG fusion proteins included Lpt1 (lipid transfer protein 1, G217B_7495), Fah1 (fumarylacetoacetate hydrolase, G217B_2697), Fdo1 (FAD-dependent oxidoreductase, G217B_6054), Ech1 (enoyl-CoA hydratase, G217B_5165), Pot1 (3-ketoacyl-CoA thiolase, G217B_8788), Cdo1 (cysteine dioxygenase, G217B_7766), Cyb2 (L-lactate cytochrome c oxidoreductase, G217B_8374), Icl1 (isocitrate lyase, G217B_1998), and Wsc1 (woronin sorting complex protein, G217B_5352).

## Data Availability

Proteomics data are available through PRIDE (Proteomics IDEntification Database) as part of the ProteomeXchange under accession PXD076035. All *Histoplasma* strains and other data are fully available without restriction upon request of the corresponding author.

## SUPPLEMENTAL MATERIAL

Figure S1 mbio.00771-26-s0001.pdf Select non-canonical PTS1 motifs are sufficient to drive peroxisomal protein localization in *Histoplasma*.

Table S1 mbio.00771-26-s0002.xlsx Unprocessed proteomics data.

Table S2 mbio.00771-26-s0003.xlsx Peroxisome-enriched proteins in PEX(+) *Histoplasma* yeast.

Table S3 mbio.00771-26-s0004.xlsx Differential abundance statistics.

Table S4 mbio.00771-26-s0005.xlsx Differential abundance statistics.

Table S5 mbio.00771-26-s0006.xlsx Combined peroxisome proteome of *Histoplasma* yeasts.

Table S6 mbio.00771-26-s0007.pdf *Histoplasma capsulatum* strains.

ASM does not own the copyrights to Supplemental Material that may be linked to, or accessed through, an article. The authors have granted ASM a non-exclusive, world-wide license to publish the Supplemental Material files. Please contact the corresponding author directly for reuse.
