# Supplementary material for: Determination of the peroxisomal proteome of pathogenic stage Histoplasma capsulatum
Source: mBio. 2026 Jun 9;17(7):e00771-26. doi: 10.1128/mbio.00771-26 (PMC13343957; doi:10.1128/mbio.00771-26)
Supplement: Figure S1 — Select non-canonical PTS1 motifs are sufficient to drive peroxisomal protein localization in Histoplasma. [file mbio.00771-26-s0001.pdf]

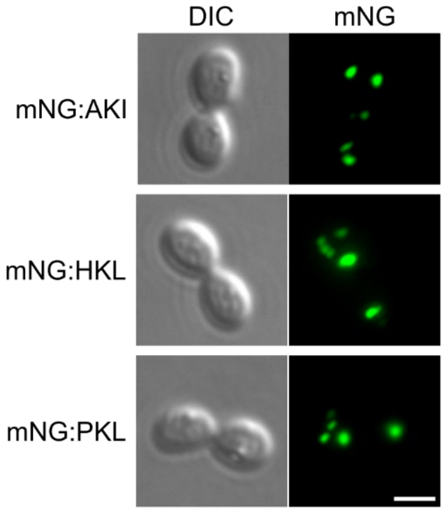

Figure S1: Select non-canonical PTS1 motifs are sufficient to drive peroxisomal protein localization in *Histoplasma.* Differential interference contrast (DIC) and fluorescence microscopy images of *Histoplasma* yeasts expressing mNeonGreen with alternative PTS1 tripeptides -AKI, -HKL, or -PKL at the C-terminus. Panels show representative images of visualized yeasts (n>50 yeast analyzed). Scale bar represents 2.5μm.
