# Supplementary material for: Determination of the peroxisomal proteome of pathogenic stage Histoplasma capsulatum
Source: mBio. 2026 Jun 9;17(7):e00771-26. doi: 10.1128/mbio.00771-26 (PMC13343957; doi:10.1128/mbio.00771-26)
Supplement: Table S6 — Histoplasma capsulatum strains. [file mbio.00771-26-s0007.pdf]

Table S6: *Histoplasma capsulatum* strains

Strain	Genotype[1,2]
WU15	*G217B ura5-42Δ*
OSU445	*G217B ura5-42Δ pex5-2Δ*
OSU614	*G217B ura5-42Δ zzz::pPB853 (URA5, P_{TEF1}-mNG)*
OSU641	*G217B ura5-42Δ pex7-1Δ*
OSU642	*G217B ura5-42Δ zzz::pPB66 (URA5, P_{TEF1}-mNG:TurboID)*
OSU643	*G217B ura5-42Δ zzz::pPB65 (URA5, P_{TEF1}-mNG:TurboID:PTS1)*
OSU675	*G217B ura5-42Δ pex5-2Δ zzz::pPB64 (URA5, P_{TEF1}-PTS2:mNG:TurboID)*
OSU676	*G217B ura5-42Δ pex5-2Δ zzz::pPB65 (URA5, P_{TEF1}-mNG:TurboID:PTS1)*
OSU677	*G217B ura5-42Δ pex5-2Δ zzz::pPB66 (URA5, P_{TEF1}-mNG:TurboID)*
OSU678	*G217B ura5-42Δ pex7-1Δ zzz::pPB64 (URA5, P_{TEF1}-PTS2:mNG:TurboID)*
OSU679	*G217B ura5-42Δ pex7-1Δ zzz::pPB65 (URA5, P_{TEF1}-mNG:TurboID:PTS1)*
OSU680	*G217B ura5-42Δ pex7-1Δ zzz::pPB66 (URA5, P_{TEF1}-mNG:TurboID)*
OSU671	*G217B ura5-42 Δ zzz::pPB67 (URA5, P_{TEF1}-mNG:TurboID:$PTS1_{Sid3\ 3AAs}$)*
OSU672	*G217B ura5-42 Δ zzz::pPB68 (URA5, P_{TEF1}-mNG:TurboID:$PTS1_{Sid3\ 9AAs}$)*
OSU673	*G217B ura5-42 Δ zzz::pPB69 (URA5, P_{TEF1}-mNG:TurboID:$PTS1_{Sid3\ 20AAs}$)*
OSU698	*G217B ura5-42Δ zzz::pPB74 (URA5, P_{TEF1}-mNG:$PTS1_{CatP}$)*
OSU699	*G217B ura5-42Δ zzz::pPB75 (URA5, P_{TEF1}-mNG:$PTS1_{Sid1}$)*
OSU700	*G217B ura5-42Δ zzz::pPB76 (URA5, P_{TEF1}-mNG:$PTS1_{Sid3}$)*
OSU737	G217B ura5-42Δ pex7-1Δ pex5-3Δ
PJB001	*G217B ura5-42Δ zzz::pPB43 (URA5, P_{TEF1}-ICL1:mNG)*
PJB002	*G217B ura5-42Δ pex5-2Δ zzz::pPB43 (URA5, P_{TEF1}-ICL1:mNG)*
PJB003	*G217B ura5-42Δ pex7-1Δ zzz::pPB43 (URA5, P_{TEF1}-ICL1:mNG)*
PJB004	*G217B ura5-42Δ zzz::pPB44 (URA5, P_{TEF1}-mNG:ICL1)*
PJB005	*G217B ura5-42Δ pex5-2Δ zzz::pPB44 (URA5, P_{TEF1}-mNG:ICL1)*
PJB006	*G217B ura5-42Δ pex7-1Δ zzz::pPB44 (URA5, $_{PTEF1}$-mNG:ICL1)*
PJB007	*G217B ura5-42Δ zzz::pPB47 (URA5, P_{TEF1}-mNG:FAH1)*
PJB008	*G217B ura5-42Δ pex5-2Δ zzz::pPB47 (URA5, $_{PTEF1}$-mNG:FAH1)*
PJB009	*G217B ura5-42Δ pex7-1Δ zzz::pPB47 (URA5, P_{TEF1}-mNG:FAH1)*
PJB010	*G217B ura5-42Δ zzz::pPB52 (URA5, P_{TEF1}-ECH1:mNG)*
PJB011	*G217B ura5-42Δ pex5-2Δ zzz::pPB52 (URA5, P_{TEF1}-ECH1:mNG)*
PJB012	*G217B ura5-42Δ pex7-1Δ zzz::pPB52 (URA5, P_{TEF1}-ECH1:mNG)*
PJB016	*G217B ura5-42Δ zzz::pPB56 (URA5, P_{TEF1} POT1:mNG)*
PJB017	*G217B ura5-42Δ pex5-2Δ zzz::pPB56 (URA5, P_{TEF1}-POT1:mNG)*
PJB018	*G217B ura5-42Δ pex7-1Δ zzz::pPB56 (URA5, P_{TEF1}-POT1:mNG)*
PJB019	*G217B ura5-42Δ zzz::pPB57 (URA5, P_{TEF1}-mNG:FDO1)*
PJB020	*G217B ura5-42Δ pex5-2Δ zzz::pPB57 (URA5, P_{TEF1}-mNG:FDO1)*
PJB021	*G217B ura5-42Δ pex7-1Δ zzz::pPB57 (URA5, P_{TEF1}-mNG:FDO1)*
PJB022	*G217B ura5-42Δ zzz::pPB58 (URA5, P_{TEF1}-WSC1:mNG)*
PJB023	*G217B ura5-42Δ pex5-2Δ zzz::pPB58 (URA5, P_{TEF1}-WSC1:mNG)*
PJB024	*G217B ura5-42Δ pex7-1Δ zzz::pPB58 (URA5, P_{TEF1}-WSC1:mNG)*

PJB025	*G217B ura5-42Δ zzz::pPB59 (URA5, P_{TEF1}-mNG:WSC1)*
PJB026	*G217B ura5-42Δ pex5-2Δ zzz::pPB59 (URA5, P_{TEF1}-mNG:WSC1)*
PJB027	*G217B ura5-42Δ pex7-1Δ zzz::pPB59 (URA5, P_{TEF1}-mNG:WSC1)*
PJB028	*G217B ura5-42Δ zzz::pPB60 (URA5, P_{TEF1}-CYB2:mNG)*
PJB029	*G217B ura5-42Δ pex5-2Δ zzz::pPB60 (URA5, P_{TEF1}-CYB2:mNG)*
PJB030	*G217B ura5-42Δ pex7-1Δ zzz::pPB60 (URA5, P_{TEF1}-CYB2:mNG)*
PJB031	*G217B ura5-42Δ zzz::pPB61 (URA5, P_{TEF1}-mNG:CYB2)*
PJB032	*G217B ura5-42Δ pex5-2Δ zzz::pPB61 (URA5, P_{TEF1}-mNG:CYB2)*
PJB033	*G217B ura5-42Δ pex7-1Δ zzz::pPB61 (URA5, P_{TEF1}-mNG:CYB2)*
PJB034	*G217B ura5-42Δ zzz::pPB62 (URA5, P_{TEF1}-CDO1:mNG)*
PJB035	*G217B ura5-42Δ pex5-2Δ zzz::pPB62 (URA5, P_{TEF1}-CDO1:mNG)*
PJB036	*G217B ura5-42Δ pex7-1Δ zzz::pPB62 (URA5, P_{TEF1}CDO1:mNG)*
PJB037	*G217B ura5-42Δ zzz::pPB63 (URA5, P_{TEF1}-mNG:CDO1)*
PJB038	*G217B ura5-42Δ pex5-2Δ zzz::pPB63 (URA5, P_{TEF1}-mNG:CDO1)*
PJB039	*G217B ura5-42Δ pex7-1Δ zzz::pPB63 (URA5, P_{TEF1}-mNG:CDO1)*
PJB040	*G217B ura5-42Δ zzz::pCD01 (URA5, P_{TEF1}-mNG:LPT1)*
PJB041	*G217B ura5-42Δ pex5-2Δ zzz::pCD01 (URA5, P_{TEF1}-mNG:LPT1)*
PJB042	*G217B ura5-42Δ pex7-1Δ zzz::pCD01 (URA5, P_{TEF1}-mNG:LPT1)*
PJB043	*G217B ura5-42Δ zzz::pCD03 (URA5, P_{TEF1}-mNG:ECH1)*
PJB044	*G217B ura5-42Δ pex5-2Δ zzz::pCD03 (URA5, P_{TEF1}-mNG:ECH1)*
PJB045	*G217B ura5-42Δ pex7-1Δ zzz::pCD03 (URA5, P_{TEF1}-mNG:ECH1)*
PJB048	*G217B ura5-42Δ zzz::pPB44 (URA5, P_{TEF1}-mNG:ICL1) zzz::pCS22 (hph, P_{TEF1}-td-TomatoRFP:SID1)*
PJB049	*G217B ura5-42Δ zzz::pPB44 (URA5, P_{TEF1}-mNG:WSC1) zzz::pCS22 (hph, P_{TEF1}-td-TomatoRFP:SID1)*
PJB052	*G217B ura5-42Δ zzz::pPB65 (URA5, P_{TEF1}-mNG:TurboID:PTS1) zzz::pCS22 (hph, P_{TEF1}-td-TomatoRFP:SID1)*
PJB053	*G217B ura5-43Δ zzz::pCR942 (URA5, PTEF1-mNeonGreen:AKI)*
PJB054	*G217B ura5-43Δ zzz::pCR943 (URA5, PTEF1-mNeonGreen:HKL)*
PJB055	*G217B ura5-43Δ zzz::pCR944 (URA5, PTEF1-mNeonGreen:PKL)*
PJB056	*G217B ura5-42Δ pex7-1Δ pex5-3Δ zzz::pPB43 (URA5, P_{TEF1}-ICL1:mNG)*
PJB057	*G217B ura5-42Δ pex7-1Δ pex5-3Δ zzz::pPB44 (URA5, P_{TEF1}-mNG:ICL1)*

[1] all strains derived from the clinical isolate G217B (ATCC 26032).
[2] gene designations (loss of function mutants of *Histoplasma* genes in lowercase)

CATP = peroxisome catalase
hph = hygromycin phosphotransferase
SID1 = l-ornithine N(5)-oxygenase
SID3 = N(5)-hydroxyornithine:cis-anhydromevalonyl coenzyme A-N(5)-transacylase
mNG = monomeric NeonGreen fluorescent protein-encoding
PEX5 = peroxin-5 (cytosolic receptor for PTS1)
PEX7 = peroxin-7 (cytoxolic receptor for PTS2)
PTS1 = type 1 peroxisome targeting sequence tripeptide
PTS2 = type 2 peroxisome targeting sequence
TEF1 = translation elongation factor EF-1 alpha
td-Tomato = tandem-dimer Tomato red fluorescence protein
TurboID = biotin ligase derived from BioID
URA5 = orotate phosphoribosyltransferase
ICL1 = isocitrate lyase

FAH1 = fumarylacetoacetate hydrolase
ECH1 = enoyl-CoA hydratase/isomerase family protein
POT1 = 3-ketoacyl-coA thiolase
FDO1 = FAD-dependent oxidoreductase
WSC1 = woronin sorting complex protein
CYB2 = L-lactate ferricytochrome c oxidoreductase
CDO1 = cysteine dioxygenase
LPT1 = lipid transfer protein
